# AI-driven quadruped robots: from fundamental locomotion to advanced biomimetic behaviors

**DOI:** 10.3389/fnbot.2026.1855550

**Published:** 2026-07-23

**Authors:** Likai Wu, Chee-Onn Chow, Wei Ru Wong, Joon Huang Chuah, Jeevan Kanesan, Meina Zhang

**Affiliations:** 1 Department of Electrical Engineering, Faculty of Engineering, Universiti Malaya, Lembah Pantai, Kuala Lumpur, Malaysia; 2 School of Electrical Engineering, Shandong Huayu University of Technology, Dezhou, China; 3 Department of Naval Architecture and Ocean Engineering, Pusan National University, Busan, Republic of Korea

**Keywords:** artificial intelligence, energy optimization, human-robot interaction, intelligent behaviors, locomotion control, quadruped robots

## Abstract

Quadruped robots have attracted increasing attention because they can traverse uneven terrain, support field deployment, and perform tasks that are difficult for wheeled or tracked platforms. Recent advances in artificial intelligence (AI) have further expanded their capabilities from manually designed gait control toward learning-based locomotion, perception-aware adaptation, dynamic motion skills, autonomous recovery, manipulation, energy-aware operation, fault diagnosis, and human–robot interaction. However, the literature on AI-driven quadruped robotics is distributed across diverse technical topics, robot platforms, validation settings, and performance metrics, making it difficult to assess the maturity and practical value of different approaches. To address this need, this review provides an AI-centered and deployment-oriented overview of quadruped robotics. A systematic literature search was conducted using Web of Science, IEEE Xplore, ACM Digital Library, ScienceDirect, and SpringerLink, covering studies published approximately from 2000 to 2025. After screening and eligibility assessment, 287 studies were included for detailed review. The review first examines AI-driven locomotion, including reinforcement learning, non-RL machine-learning methods, model-based approaches, and hybrid strategies, with attention to robustness, sim-to-real transfer, sensor use, computational requirements, and hardware validation. It then summarizes AI-supported advanced behaviors, including jumping, fall prevention and recovery, and object manipulation, focusing on reported quantitative performance, impact management, and reliability. Finally, it discusses system-level topics that affect real-world deployment, including fault diagnosis, energy-efficient control, shared autonomy, trust-aware and explainable interaction, and safety-aware human–robot collaboration. By organizing the literature according to robot capabilities, validation maturity, and deployment challenges, this review helps clarify the current progress, limitations, and future directions of AI-driven quadruped robots.

## 1 Introduction

Quadruped robots, designed to mimic the locomotion of four-legged animals (Ijspeert, 2008), have garnered significant attention in the field of robotics due to their unique advantages in versatility, stability, and ability to navigate complex terrains. Unlike traditional wheeled or tracked robots, quadruped robots offer a higher degree of mobility, particularly in environments that are difficult for other robot types to navigate, such as uneven or rough surfaces (Fukuoka et al., 2003). Their inherent stability, achieved through their four-legged design, allows them to perform tasks with greater reliability and resilience, making them ideal for applications ranging from disaster zone exploration to military reconnaissance, and even for assisting search and rescue operations in environments where human presence is dangerous (Shafiee et al., 2024). The capability to move efficiently across various terrains, coupled with their agility and adaptability, has positioned quadruped robots as a promising solution to real-world challenges.

In the field of AI, significant advancements in recent years have further propelled the development of quadruped robots, enhancing their performance and expanding their capabilities (Tsounis et al., 2020; Yang et al., 2020a). AI-driven technologies, such as deep learning (Villarreal et al., 2020) and reinforcement learning (Agarwal et al., 2023), have played a pivotal role in enhancing the behavior and functionality of quadruped robots. These technologies enable robots to learn from their experiences, adapt to dynamic environments, and perform tasks autonomously with minimal human intervention (Duan et al., 2016).

An important emerging direction is vision–language–action (VLA) modeling, which links natural-language instructions, visual scene understanding, and robot action generation within a common embodied-AI framework. General robotics studies such as SayCan, PaLM-E, and RT-2 have shown how language-conditioned reasoning, large-scale multimodal learning, and embodied policies can support more general robot decision-making and task execution (Ahn et al., 2022; Driess et al., 2023; Zitkovich et al., 2023). For quadruped robots, VLA is particularly relevant because high-level commands such as inspecting a room, avoiding an obstacle, or approaching an object must be grounded in terrain geometry, traversability, body stability, contact timing, and actuator limits. Recent quadruped-oriented systems, including CognitiveDog, QUAR-VLA, and NaVILA, illustrate early attempts to connect language instructions and visual observations with navigation, locomotion, and manipulation actions on legged platforms (Lykov et al., 2024; Ding et al., 2023; Cheng et al., 2024). At the same time, VLA for quadrupeds remains less mature than low-level locomotion learning; reliable grounding, action feasibility, latency, computation load, safety constraints, and hardware validation are still major barriers. This review includes VLA as an emerging AI direction while also examining the established areas of locomotion control, dynamic behaviors, reliability, energy efficiency, and human–robot interaction.

At the heart of quadruped robotics lies the fundamental aspect of locomotion. Early studies in quadruped robots focused primarily on simple gait generation, with engineers designing algorithms that allowed robots to walk steadily on flat surfaces (Yu et al., 2022; Fujita and Kitano, 1998; Golubitsky et al., 1998; HIROSE, 1984). However, as the field progressed, researchers began to address more complex challenges, such as walking on uneven terrain, climbing obstacles, and maintaining balance during dynamic movements. These challenges require advanced control algorithms, as well as the integration of multiple AI techniques that enable the robot to learn and adapt to its environment in real-time (Muhamad et al., 2024; RAIBERT, 1990; Kimura et al., 1999).

Beyond basic locomotion, recent developments have also focused on enabling quadruped robots to perform more sophisticated actions. For example, the ability to jump has emerged as a significant milestone in robot mobility (Park et al., 2015). Jumping allows quadruped robots to traverse obstacles that are too high for them to walk over, increasing their overall operational versatility. Similarly, advancements in fall prevention and recovery mechanisms have enabled quadruped robots to avoid or recover from falls, improving their robustness and reliability in real-world settings (Sun et al., 2024). In addition, quadruped robots are now being developed to manipulate objects, adding another layer of complexity and usefulness to their behavior (Jeon et al., 2024).

AI technologies also play a critical role in improving the reliability and efficiency of quadruped robots through self-diagnosis and energy optimization. AI-based self-diagnosis systems have been implemented to detect and address issues before they become critical, allowing the robot to function autonomously (Liling et al., 2015). Furthermore, AI-driven optimization techniques are being applied to enhance energy efficiency, a critical factor in extending the robot's operational duration, especially in tasks that require extended mobility, such as exploration or search and rescue missions (Yan et al., 2024b). Besides, The integration of AI also enhances human-robot interaction, making quadruped robots more intuitive and effective in collaborative environments (Shen et al., 2024).

**Figure 1:**
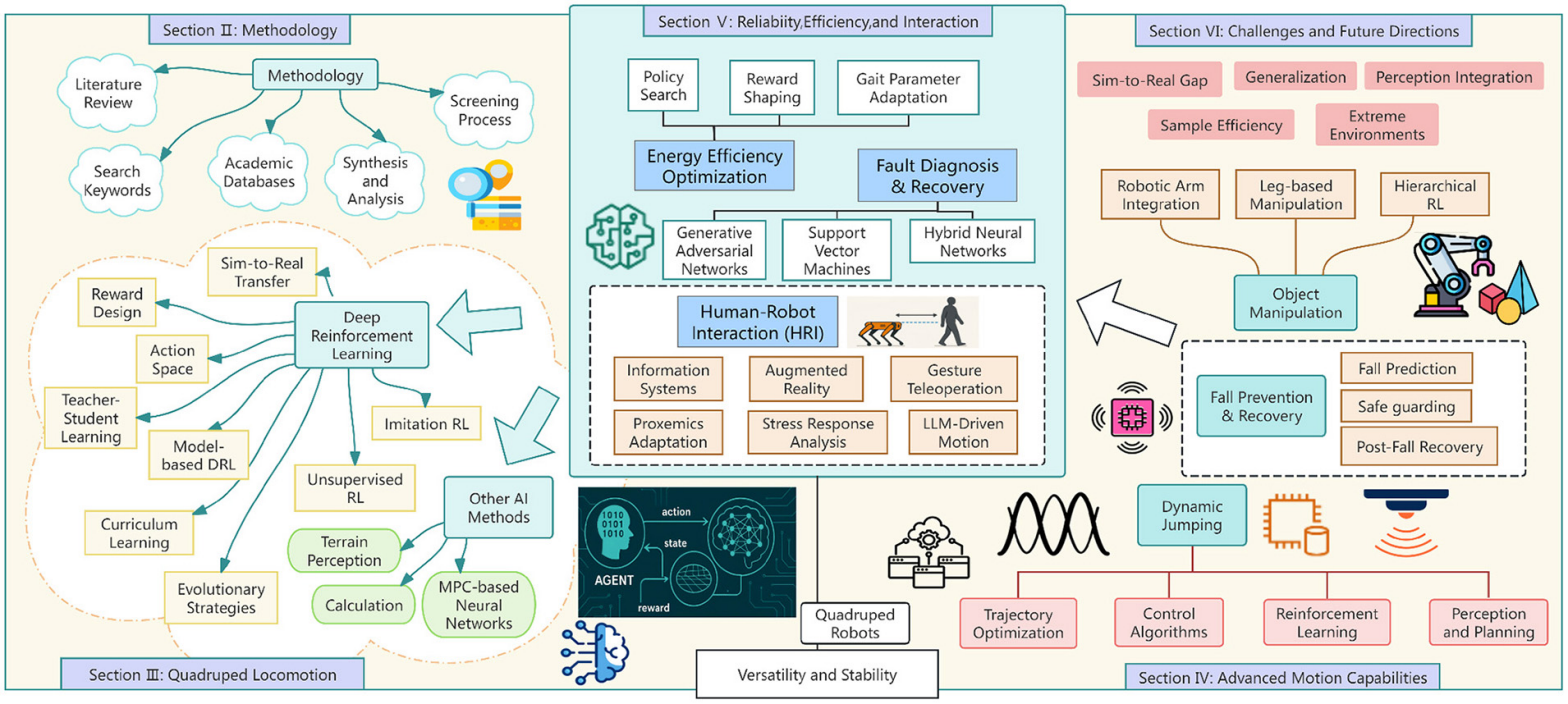
Overview of this review on AI-driven quadruped robots, showing the main framework from methodology to advanced capabilities. It illustrates the flow from literature analysis through AI-driven locomotion, to advanced behaviors including jumping, fall recovery, and object manipulation, and further to system-level aspects such as fault diagnosis, energy efficiency, and human-robot interaction, highlighting links to challenges and future directions.

This review paper begins with the fundamental aspect of locomotion, then examines more advanced and intelligent behaviors. The main content of this review paper is shown in Figure 1.

### 1.1 Related review papers

Since the development of quadruped robots, numerous review articles have been published. In this study, we selected 12 representative review papers and conducted a comparative analysis of their content.

He et al. (2019) focused on terrain adaptability, including slope walking and disturbance handling strategies. Zhong et al. (2019) classified quadruped legs into three structural types and analyzes their mechanical advantages. Fukuhara et al. (2022) compared anatomical features of animals and robots to understand multifunctional body part behaviors. In terms of control, Hamrani et al. (2025) highlighted the evolution from traditional methods to reinforcement learning, emphasizing sensor fusion. Zhao et al. (2023) reviewed motion planning and compliance control strategies, while Kotha et al. (2024) compared VMC, MPC, and RL for unstructured environments. He and Gao (2020) surveyed hardware advances in highly dynamic legged robots, including novel actuators and leg designs. Torres-Pardo et al. (2022) focused on locomotion performance across humans and robots on irregular terrain. Foundational works (Ijspeert, 2008; QiDi et al., 2009) introduced biologically inspired CPG models for robot locomotion. Structural and gait design developments were reviewed in Biswal and Mohanty (2021) and Taheri and Mozayani (2023).

### 1.2 Purpose of this review

In recent years, artificial intelligence has become deeply embedded in quadruped robotics. Its role is no longer limited to improving a walking controller; learning-based and data-driven methods are now used in terrain perception, policy adaptation, dynamic skills, fault detection, energy-aware control, and human–robot interaction. This expansion has made the field productive but also difficult to read as a whole. Studies often report results on different robot platforms, in different validation settings, and with different performance indicators, so it is not easy to judge which methods are mature, which remain mainly simulation demonstrations, and which technical barriers still limit deployment.

This review provides a deployment-oriented map of AI-driven quadruped robotics. The literature is organized by robot capability, covering locomotion, jumping, fall prevention and recovery, object manipulation, fault diagnosis, energy efficiency, and human–robot interaction. Within these topics, the review compares major algorithmic choices, including model-free RL, model-based and hybrid methods, and non-RL machine-learning approaches, while also considering validation level, sensor use, robustness, computation, and reported quantitative performance. This organization helps clarify how AI is currently used in quadruped systems, what evidence supports each type of method, and where open problems remain, including sim-to-real transfer, actuator power density, battery endurance, thermal effects, repeated-fall reliability, and multimodal perception–action integration. Table 1 summarizes the relationship between this review and representative earlier reviews.

**Table 1:** Comparison of the current review with other relevant reviews.

Reviews	Ours	R1	R2	R3	R4	R5	R6
(a)							
Year of publication	2026	2025	2024	2023	2023	2022	2022
• Review methodology	✓	✓	✓	X	X	✓	X
• Year-wise distribution	✓	✓	X	X	X	X	X
• Machine learning	✓	✓	✓	✓	✓	✓	✓
• Deep learning	✓	✓	✓	✓	✓	✓	✓
• Jumping	✓	X	✓	X	X	X	✓
• Fall recovery	✓	✓	X	✓	X	X	X
• Object manipulation	✓	X	X	X	X	✓	X
• Fault diagnosis	✓	X	✓	X	X	X	X
• Energy efficiency	✓	✓	X	✓	✓	X	X
• Human–robot interaction	✓	✓	X	X	X	X	X
• Comparative analysis	✓	✓	X	✓	✓	✓	✓
**Reviews**	**Ours**	**R7**	**R8**	**R9**	**R10**	**R11**	**R12**
(b)							
Year of publication	2026	2021	2020	2019	2019	2009	2008
• Review methodology	✓	X	X	X	X	X	X
• Year-wise distribution	✓	X	X	X	X	X	X
• Machine learning	✓	X	✓	✓	✓	X	X
• Deep learning	✓	✓	✓	✓	✓	X	X
• Jumping	✓	X	X	X	X	X	X
• Fall recovery	✓	✓	X	X	X	X	X
• Object manipulation	✓	X	✓	X	X	X	X
• Fault diagnosis	✓	X	X	X	X	X	X
• Energy efficiency	✓	X	X	✓	X	X	X
• Human–robot interaction	✓	X	X	X	X	X	X
• Comparative analysis	✓	✓	✓	X	✓	✓	✓

R1 = Hamrani et al. (2025); R2 = Kotha et al. (2024); R3 = Taheri and Mozayani (2023); R4 = Zhao et al. (2023); R5 = Torres-Pardo et al. (2022); R6 = Fukuhara et al. (2022).

R7 = Biswal and Mohanty (2021); R8 = He and Gao (2020); R9 = He et al. (2019); R10 = Zhong et al. (2019); R11 = QiDi et al. (2009); R12 = Ijspeert (2008).

A checkmark indicates that the review contains a substantive discussion of the item rather than only a brief mention. Review methodology refers to explicit database/search/screening procedures; comparative analysis refers to direct comparison among methods, platforms, or evidence types.

To keep the comparison in Table 1 consistent, each category follows an operational definition. “Review Methodology” refers to explicit reporting of databases, search terms, and selection criteria. “Machine Learning” and “Deep Learning” refer to dedicated discussion of learning-based models rather than isolated examples. “Comparative Analysis” refers to direct contrast among methods, validation settings, or performance indicators.

The section arrangement of this paper is as follows: Section 1 introduces the significance of quadruped robots and the impact of AI on enhancing their capabilities, and compares previous review articles on quadruped robotics. Section 2 describes the methodology adopted in conducting this review paper. Section 3 focuses on AI-driven locomotion, detailing reinforcement learning techniques and other AI algorithms. Section 4 explores advanced behaviors like jumping, fall prevention, and object manipulation. Section 5 discusses AI-based fault diagnosis, energy efficiency improvements and human-robot interaction enhancements. Finally, Section 6 outlines challenges and future directions.

**Figure 2:**
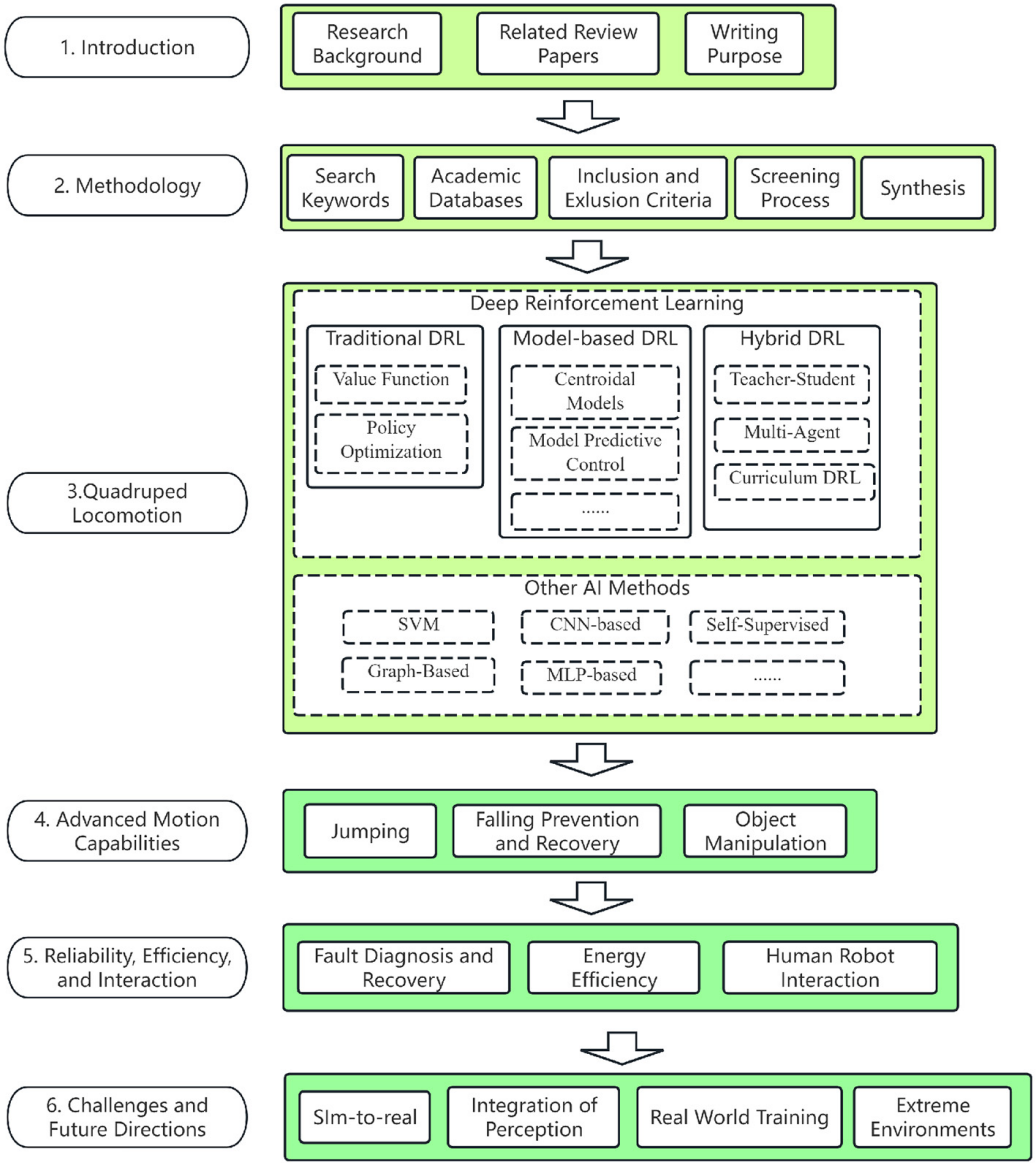
Overview of the structure of this review paper.

The structure of this review paper is shown in Figure 2.

## 2 Methodology

This section outlines the systematic methodology adopted for conducting this review. The procedure was designed to improve reproducibility, transparency, and interpretability of the 287 selected studies.

### 2.1 Search strategy and boolean queries

The search covered publications from 2000 to 2026. The search strategy combined broad AI-quadruped terms with application-specific terms. The core Boolean query was:

(“quadruped robot*” OR “quadrupedal robot*” OR “legged robot*”) AND (“artificial intelligence” OR “machine learning” OR “deep learning” OR “reinforcement learning” OR “neural network*” OR “imitation learning” OR “vision-language-action” OR “multimodal model*”)

To capture specific functional areas, the following application query was also used:

(“quadruped robot*” OR “quadrupedal robot*”) AND (“jump*” OR “fall prevention” OR “fall recovery” OR “self-righting” OR “object manipulation” OR “loco-manipulation” OR “fault diagnosis” OR “fault-tolerant” OR “energy efficiency” OR “cost of transport” OR “human-robot interaction” OR “shared autonomy”)

For reinforcement-learning methods, an additional algorithm-specific query was used:

(“quadruped robot*” OR “legged locomotion”) AND (“PPO” OR “proximal policy optimization” OR “SAC” OR “soft actor-critic” OR “TD3” OR “twin delayed deep deterministic policy gradient” OR “DDPG” OR “deep deterministic policy gradient” OR “model predictive control” OR “model-based reinforcement learning”)

The search was performed across Web of Science, IEEE Xplore, ACM Digital Library, ScienceDirect, and SpringerLink.

### 2.2 Screening process

To ensure the relevance and quality of the reviewed literature, inclusion and exclusion criteria were established before the screening process. Articles were included if they met the following conditions:

- they focused on quadruped or closely related legged robotic systems;
- they included AI, machine learning, reinforcement learning, perception, adaptive control, or data-driven diagnosis/interaction components;
- they provided empirical data, simulation results, hardware experiments, theoretical analysis, or a clearly described system architecture;
- they were published in peer-reviewed journals, reputable conferences, or authoritative academic books.

Studies were excluded if they were purely conceptual with no experimental, simulation, or analytical validation; tangentially related to quadruped robotics; duplicate records; inaccessible full texts; or papers with insufficient methodological detail for comparison.

**Figure 3:**
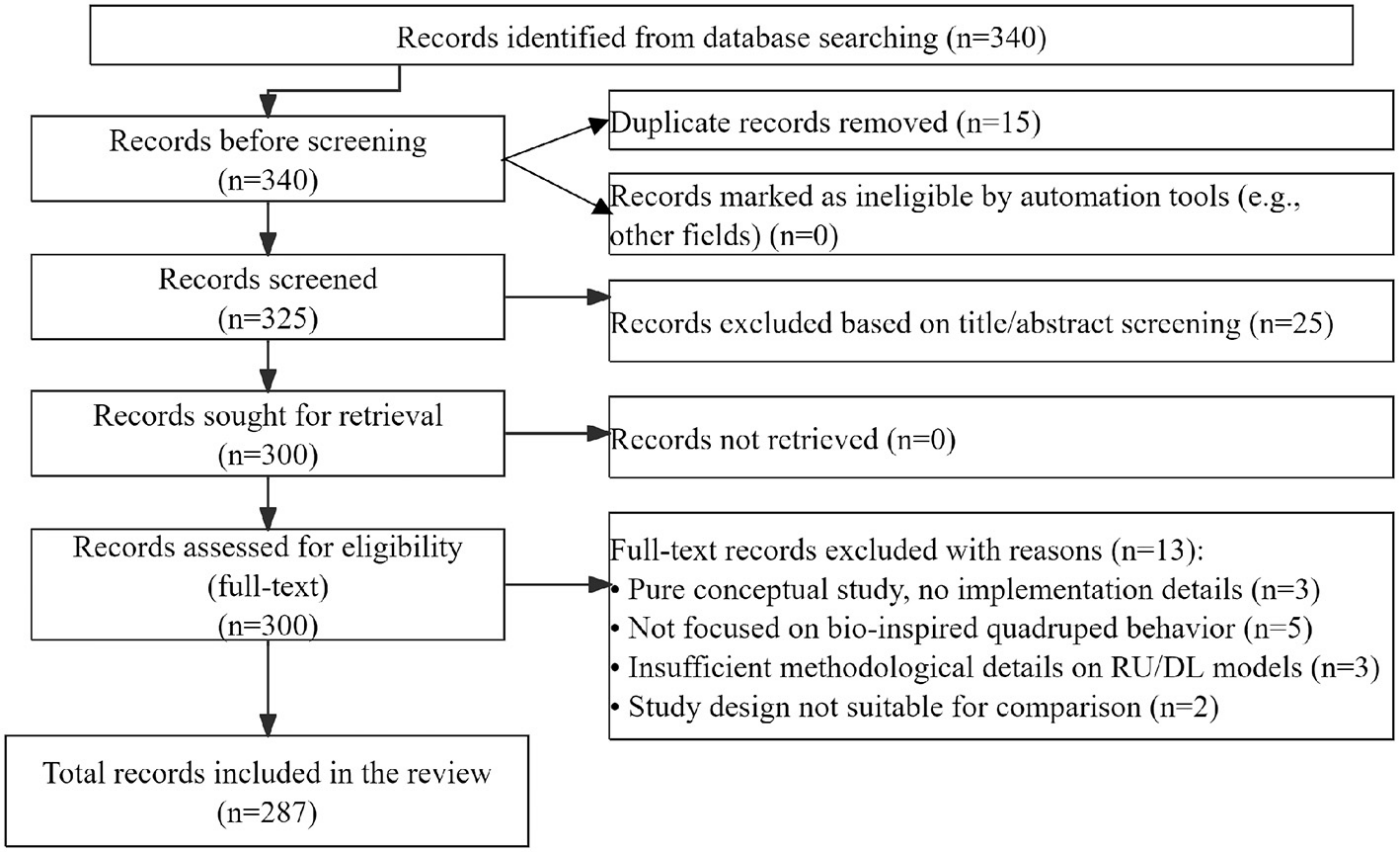
Methodological framework of paper selection for systematic review.

The methodological framework of paper selection for systematic review is shown in Figure 3.

### 2.3 Quality assessment and evidence coding

We used a categorical quality-assessment framework. Each included study was coded along the dimensions in Table 2. The framework helps readers interpret the maturity of each approach, especially when comparing simulation-only papers with hardware-validated or field-deployed systems.

**Table 2:** Quality-assessment and evidence-maturity framework used for the systematic synthesis.

Dimension	Coding levels	Interpretation in this review
Validation maturity	V0: simulation/theory only; V1: controlled hardware experiment; V2: field or long-duration real-world deployment	Distinguishes algorithms demonstrated only in simulators from those tested on physical quadrupeds and those evaluated in realistic deployment conditions.
Reproducibility	R0: insufficient parameters; R1: enough implementation details for partial reproduction; R2: code, data, benchmark, or detailed supplementary material available	Indicates whether readers can reproduce the method, compare baselines, or reuse datasets and trained models.
Robustness assessment	B0: nominal-condition test only; B1: disturbances or terrain variation included; B2: repeated trials, multi-terrain tests, payload/fault variation, or sim-to-real stress tests	Captures whether performance is demonstrated under uncertainty and repeated operation rather than a single ideal trial.
Quantitative reporting	Q0: qualitative only; Q1: one main metric; Q2: multiple metrics such as success rate, tracking error, energy, speed, computation time, or recovery time	Reflects the extent to which the study supports comparative synthesis.
Deployment relevance	D0: isolated controller or module; D1: integrated robot behavior; D2: task-level autonomy involving perception, planning, interaction, or field constraints	Indicates how closely the work approaches practical quadruped operation.

## 3 Quadruped locomotion

**Figure 4:**
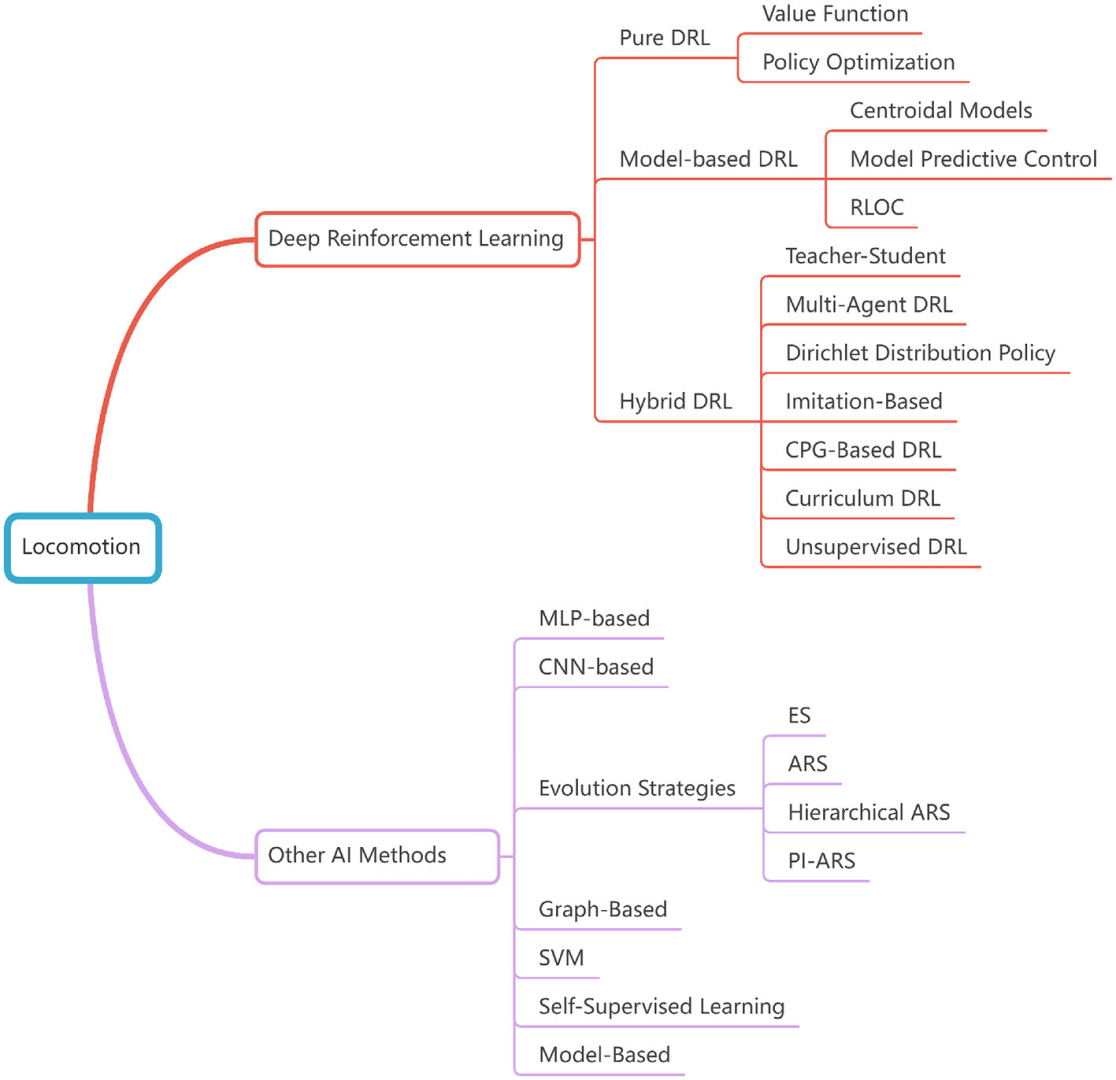
Taxonomy of AI algorithms for quadruped robots.

Achieving robust and adaptive locomotion is the foundational challenge for quadruped robots. Early quadrupeds relied on carefully engineered controllers and gait patterns, while newer systems increasingly leverage data-driven learning to attain agility. In this section, we first provide an in-depth survey of techniques and variations within deep reinforcement learning (DRL) that have been explored to push quadrupedal locomotion performance. We then outline other AI approaches which also significantly contribute to quadruped locomotion capabilities. Figure 4 presents the taxonomy of the AI algorithms for quadruped robots.

### 3.1 Deep reinforcement learning methods

In the past decade, DRL has revolutionized locomotion control of quadruped robots (Khan et al., 2024; Li et al., 2023a; Zhang et al., 2024d; Peng et al., 2017; Mnih et al., 2015; Wang et al., 2016; Heess et al., 2017; Xu et al., 2024). DRL enables robots to learn gait policies through trial-and-error interactions, rather than relying purely on pre-programmed models. Pioneering works demonstrated that with proper training, DRL policy can coordinate a quadruped's 12+ joints to walk, turn, or recover balance in ways not achievable with manual tuning (Zhang et al., 2022). However, applying DRL to real robots raises several challenges: how to gather sufficient experience safely, how to bridge the sim-to-real gap, how to design reward functions that induce desired gaits, and how to ensure stability and efficiency of the learned behavior. Over the years, researchers have proposed a wide spectrum of techniques to address these challenges, which can broadly be grouped into themes such as reward shaping, action space design, sim-to-real transfer, model-based enhancements, and specialized learning paradigms.

#### 3.1.1 Comparative perspective on major RL algorithms

A clearer comparison of major RL algorithm families is useful because quadruped locomotion papers often report results using different learning paradigms, reward designs, simulators, and hardware platforms. Table 3 summarizes the main practical trade-offs among PPO, SAC, TD3/DDPG, model-based or MPC-assisted learning, and gradient-free optimization. In broad terms, PPO is widely adopted for large-scale simulation training because of its stability and ease of parallelization, but it is relatively sample intensive. SAC and related off-policy algorithms can be more sample efficient and are attractive for continuous-control tasks such as jumping or manipulation, but they are sensitive to reward scaling and entropy tuning. TD3 and DDPG provide deterministic actor–critic baselines for continuous control and energy-aware optimization, but they can be brittle when exploration is difficult or the value function is poorly estimated. Model-based and hybrid methods reduce sample requirements and improve interpretability by injecting dynamics knowledge, but their performance depends on model fidelity. These differences make direct numerical comparison difficult unless studies report common metrics such as success rate, velocity tracking error, cost of transport, power consumption, terrain-disturbance tolerance, and real-time inference cost.

**Table 3:** Comparison of major RL and optimization paradigms used in quadruped locomotion and advanced behaviors.

Paradigm	Typical learning style	Strengths	Limitations	Typical role in quadruped robotics
PPO (Schulman et al., 2017)	On-policy stochastic actor–critic	Stable under large-scale parallel simulation; robust with domain randomization and privileged learning	Sample intensive; repeated simulation rollouts are required; real-hardware training is costly	Robust locomotion policies, rough-terrain walking, teacher–student pipelines, and sim-to-real locomotion.
SAC (Haarnoja et al., 2018b)	Off-policy maximum-entropy actor–critic	Better sample reuse; encourages exploration; suitable for continuous actions	Sensitive to entropy and reward scaling; more complex training dynamics	Jumping, manipulation, and adaptive locomotion where exploration and continuous torque/position control are central.
TD3/DDPG (Fujimoto et al., 2018; Lillicrap et al., 2016)	Off-policy deterministic actor–critic	Efficient for continuous control; useful for energy-aware or tracking-oriented objectives	Exploration can be brittle; value overestimation and reward sensitivity require careful tuning	Gait-parameter adaptation, energy minimization, and controller refinement.
Model-based RL / MPC hybrids	Dynamics-informed learning and online optimization	More interpretable; improved sample efficiency; can encode contact, actuator, and safety constraints	Requires model identification; may be sensitive to unmodeled contacts, latency, and actuator saturation	Terrain-aware locomotion, hardware-constrained jumping, robust tracking, and safe recovery (Xie et al., 2022; Gangapurwala et al., 2022; Da et al., 2021).
ES / ARS / random search	Gradient-free policy optimization	Simple, scalable, and less dependent on differentiability	Often needs many evaluations; less efficient for high-dimensional perception inputs	Baseline policy search and visual-locomotion acceleration when gradients or accurate value functions are unreliable (Salimans et al., 2017; Rajeswaran et al., 2017; Mania et al., 2018).

#### 3.1.2 Reward function design and tuning

The choice of reward function in DRL largely determines the gait that is learned. Recent works have put emphasis on reward shaping and augmentation to guide learning. For instance, researchers often include penalty terms for excessive joint torques, collisions, or body tilt to promote stable walking (Yang et al., 2020a; Bharadhwaj et al., 2021; Lee et al., 2020). Such methods help steer the policy toward physically sensible motions. In addition, instead of hand-crafting rewards, another trend is leveraging imitation learning: the agent is rewarded for mimicking reference motions from either motion capture of animals or trajectories from a planner. For example, Peng et al. (2020) used motion clips of real dogs to train a policy via imitation, resulting in remarkably natural trotting and galloping gaits. By integrating imitation rewards, the DRL agent can acquire complex behaviors more quickly and realistically than using sparse task rewards alone (Peng et al., 2018a; Shao et al., 2024; Kim et al., 2024).

To efficiently acquire these skills, specialized learning paradigms are employed. Curriculum Learning structures training progressively, starting with simple tasks before advancing to complex ones, which mirrors animal learning and prevents early failures (Zuo et al., 2024; Luo et al., 2024a; Yan et al., 2024a). The Teacher-Student and Privileged Learning framework trains a “teacher” policy in simulation with access to privileged information, then distills this knowledge into a “student” policy that operates under real-world sensing constraints, effectively bridging the sim-to-real gap (Yan et al., 2024a; Miki et al., 2022; Margolis et al., 2024; Wu et al., 2023).

For safety-critical deployment, Constrained and Safe RL incorporates constraints into the learning process to prevent violations of safety conditions, a crucial direction for real-world training (Gangapurwala et al., 2020; Ma et al., 2024). The integration of safety constraints directly into the learning objective is arguably non-negotiable for any serious real-world deployment. It moves the field beyond pure performance optimization toward responsible and reliable autonomy, marking a critical maturation point for DRL applications in robotics.

#### 3.1.3 Sim-to-real transfer and domain randomization

**Figure 5:**
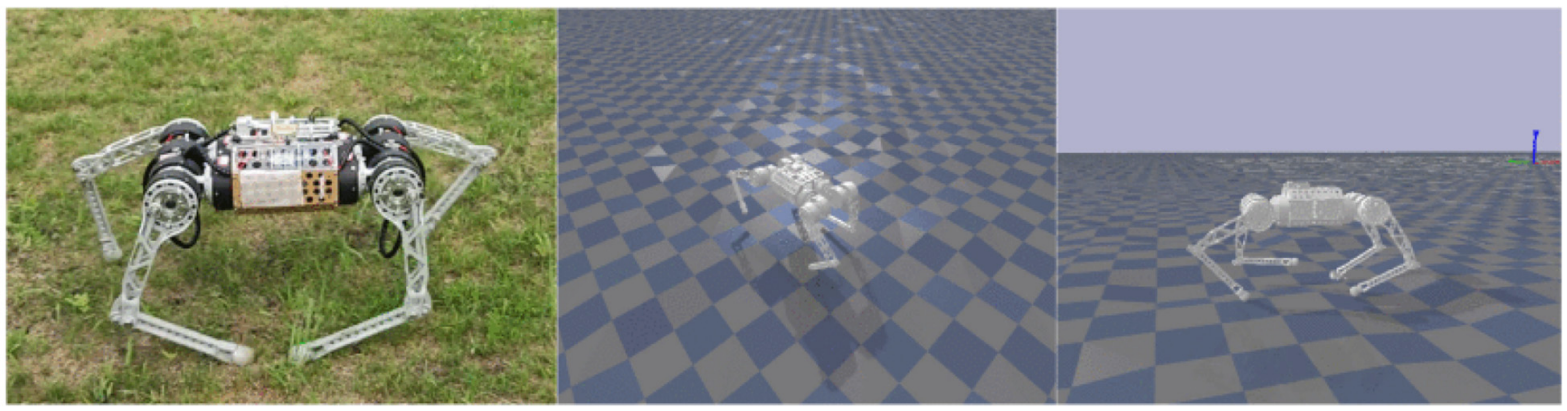
This figure shows a robotic system designed to simulate a quadruped movement. The **left** image depicts the physical robot, while the **middle** and **right** images show its virtual model in a simulation environment, demonstrating its gait and movement capabilities (these images are from Li et al., 2024b and are permitted under CC4.)

Training locomotion policies directly on hardware can be risky and slow (Haarnoja et al., 2018a; Yang et al., 2020b; Levine et al., 2018), so most studies train in physics simulators and then deploy policies on the real robot. The discrepancy between simulation and reality is a key issue known as the reality gap (Neunert et al., 2016; Boeing and Bräunl, 2012; Tian et al., 2024). Figure 5 shows a robotic system designed to simulate a quadruped movement.

Tobin et al. (2017) introduced dynamics randomization to tackle this gap: during training, the simulator's physical parameters, such as mass, friction, motor gains, latency, etc., are randomly perturbed within plausible range. This forces the policy to become robust against modeling errors. For example, Peng et al. (2018b); Xie et al. (2021) randomized robot body dimensions, motor strength, and even added artificial sensor noise, enabling a policy to succeed on a real robot despite simulator inaccuracies. Domain randomization is now standard practice, and policies have been successfully transferred to hardware with zero additional tuning by exposing them to enough simulated variability (Hwangbo et al., 2019; Kumar et al., 2021; Nagabandi et al., 2018; Feng et al., 2022; Luo et al., 2024b).

Another approach is to perform explicit system identification to make the simulator as close as possible to reality (Hanna and Stone, 2017; Tan et al., 2018). Zhu and Rosendo (2022) described calibrating an “accurate model in simulation” for their quadruped by adjusting parameters to match real-world data, and show that this greatly improves policy transfer. Unlike domain randomization, system identification seeks to narrow the sim-to-real gap by explicitly estimating physical parameters from real-world data.

Recent advances integrate online system identification with adaptive control, where the robot continually updates its dynamics model during deployment, allowing the policy to adjust in real-time to changes such as payload variation or mechanical wear (Li et al., 2024b; Bharadhwaj et al., 2021). Hybrid methods that combine domain randomization with guided parameter estimation, using Bayesian optimization or meta-learning to focus randomization on physically plausible regions, have shown improved sample efficiency and final performance (Lee et al., 2020; Peng et al., 2020). While domain randomization excels in generalization across broadly varied conditions, system identification provides a more sample-efficient and physically grounded pathway for adaptation to specific hardware instances, albeit at the cost of additional modeling and identification effort.

Integral to both strategies is the design of the action space, which defines how the policy interacts with the robot's actuators. The choice of action representation significantly influences learning efficiency, policy robustness, and sim-to-real transferability (Peng and van de Panne, 2017; Bellegarda and Byl, 2019; Zhang et al., 2023). Low-level action spaces, such as joint position, velocity, or torque commands, provide fine-grained control but often result in high-dimensional, challenging learning problems. In contrast, higher-level action abstractions can reduce the policy's search space and embed domain knowledge, thereby accelerating training and improving stability (Xie et al., 2022; Liu et al., 2024a). A particularly effective approach is the use of central pattern generator (CPG)-based or phase-based parameterizations, where the policy modulates rhythmic motion primitives rather than controlling each joint independently (Gangapurwala et al., 2022; Da et al., 2021). This biologically inspired structure can enhance robustness by ensuring gait consistency. Furthermore, hierarchical action spaces that combine high-level task commands with low-level refinement have been explored to balance expressivity and learnability (Zhang et al., 2024c). The action space design must align with the chosen sim-to-real strategy: for example, a parameterized gait representation may transfer more reliably under domain randomization, whereas a torque-based action space may benefit more from precise system identification to accurately model actuator dynamics. Recent work also investigates adaptive action spaces, where the policy or an auxiliary network learns an action representation that is optimized for both performance and transfer, blurring the line between controller design and policy learning (Bellegarda and Ijspeert, 2022; Zuo et al., 2024). Across these studies, the evidence base differs substantially. Simulation-only studies can explore large terrain distributions, reward variants, and randomization ranges, but their reported metrics do not necessarily predict hardware reliability. Hardware-validated studies are more convincing for sim-to-real transfer, especially when they report terrain type, speed range, disturbance magnitude, payload, trial count, actuator limits, and inference rate. Sensor modality is another important differentiator: proprioceptive policies are computationally efficient and robust to poor visibility, whereas vision-, LiDAR-, and tactile-enhanced policies improve anticipation of footholds and terrain properties but introduce perception latency, calibration error, and higher computation requirements. A robust comparison is most informative when it reports measurable indicators rather than only describing the controller type. Table 4 therefore reports representative numerical results from selected studies, including speed, tracking error, obstacle or step height, payload robustness, trial counts, cost of transport, power use, hardware limits, and sim-to-real evidence. Because the robot platforms, test environments, and objectives differ, these values should be interpreted as reported evidence rather than as a single standardized benchmark.

**Table 4:** Representative numerical performance reported in AI-driven quadruped locomotion studies.

Study/approach	Platform and validation	Reported numerical performance	Evidence and transfer notes
Hwangbo et al. (2019); learned actuator model + RL	ANYmal; proprioception and IMU; sim-to-real hardware transfer	Real forward speed 1.5 m/s for a 1.6 m/s command; 1.58 m/s in simulation; linear velocity error 0.143 m/s; yaw-rate error 0.174 rad/s; self-righting in < 3 s from 9 fallen configurations; average torque 8.23 Nm and mechanical power 78.1 W, compared with 11.7 Nm and 97.3 W for the baseline	Controller used on hardware for more than 3 months; high-speed policy used 40 Nm torque and 12 rad/s joint-velocity limits; about 4 h computation for 9 simulated training days
Lee et al. (2020); proprioceptive rough-terrain RL	ANYmal-B/C; joint encoders and IMU; natural terrain and DARPA field tests	Average speeds: 0.452 m/s on moss, 0.338 m/s on mud, 0.248 m/s in vegetation; step traversal 16.8 cm; with 10 kg payload (22.7% robot weight), steps up to 13.4 cm; COT 0.423/0.692/1.23 on moss/mud/vegetation	Four 60-min DARPA missions reported zero locomotion failures; heading error within 10° in lateral motion vs. 30° for the baseline
Miki et al. (2022); perceptive locomotion	ANYmal; proprioception plus LiDAR or active-stereo elevation map; field deployment	Maximum flat-ground speed 1.2 m/s vs. 0.6 m/s proprioceptive baseline; turning speed 3 rad/s vs. 0.6 rad/s baseline; reliable step traversal up to 30.5 cm; step tests covered 12–36.5 cm with 10 trials per height	Completed a 2.2 km alpine hike with 120 m elevation gain in 78 min and no fall; ascent up to 38%; four ANYmals explored more than 1700 m in DARPA tunnel, urban, and cave courses without a fall
Margolis et al. (2024); rapid locomotion via RL	MIT Mini Cheetah; simulation-trained policy with online system identification; zero-shot hardware deployment	Sustained 3.9 m/s indoor sprint; 3.4 m/s outdoor 10 m dash on grass; 5.7 rad/s indoor spin; Froude number 5.1; training completed in under 3 h on one RTX 3090 GPU	Demonstrated fast running on grass, ice, gravel, and gravel hills; domain randomization included friction 0.05–4.00, restitution 0–1.00, payload mass -1 to 3 kg, and motor strength 90–110%
Hoeller et al. (2023); hierarchical parkour learning	ANYmal; learned locomotion skills plus perception and high-level skill selection; hardware transfer	Real-world parkour navigation with speeds up to 2 m/s; skills include walking, jumping, climbing, and crouching across consecutive obstacles	Modules trained from simulation data; no expert demonstration, offline computation, or prior environment map required during deployment
Zhuang et al. (2023); vision-based parkour RL	Unitree A1 and Go1; egocentric depth camera; onboard computation and power	Climb 0.40 m obstacles (1.53 × robot height); leap 0.60 m gaps (1.5 × robot length); crawl beneath 0.20 m barriers (0.76 × robot height); squeeze through 0.28 m slits	Same system demonstrated on two low-cost quadruped platforms; skills distilled into a single vision-based parkour policy
Luo et al. (2024a); PIE parkour framework	DEEP Robotics Lite3; low-cost egocentric depth camera; zero-shot real-world deployment	Leap onto/off steps 0.75 m high (3 × robot height); negotiate 1.0 m gaps (3 × robot length); climb stairs 0.25 m high (1 × robot height); 2 km outdoor hike with 153 m elevation gain in 40 min	One-stage implicit-explicit learning framework; reported indoor and outdoor zero-shot deployment without extensive fine-tuning

The values are reported as stated in the cited papers and are not normalized across platforms, robot sizes, environments, or task objectives. The table is intended to provide concrete evidence for comparing validation maturity and performance reporting across representative studies.

#### 3.1.4 Model-based enhancements and alternative optimization

While model-free DRL is effective, it can be computationally expensive. Model-based DRL utilizes predefined environment dynamics to improve data efficiency. Common models include Centroidal Models (Xie et al., 2022) for center-of-mass dynamics, Model Predictive Control (MPC) (Liu et al., 2024a) for trajectory optimization, hybrid RL-OC model (Gangapurwala et al., 2022; Da et al., 2021), Central Pattern Generator (CPG) model (Zhang et al., 2024c; Bellegarda and Ijspeert, 2022). The trend toward model-based enhancements is a welcome and necessary correction to the initial enthusiasm for purely model-free approaches. By grounding learning in physical models, these hybrid methods offer greater sample efficiency, improved interpretability, and often more stable convergence. They represent a pragmatic fusion of classical control theory's robustness with modern learning's adaptability.

Parallel to gradient-based RL, Evolutionary Strategies and Random Search offer alternative optimization pathways. Methods like Evolution Strategies (ES) (Salimans et al., 2017; Rajeswaran et al., 2017), Augmented Random Search (ARS) (Mania et al., 2018), and its variants, e.g., combined with high-level planning (Jain et al., 2019) or predictive information (Lee et al., 2022), have been successfully applied to discover effective controllers, demonstrating the versatility of optimization approaches in locomotion (Yu et al., 2022; Song et al., 2020; Yu et al., 2020; Huang et al., 2020).

ES and ARS methods often simpler to implement and parallelize, can avoid issues like local minima and reward sensitivity inherent in gradient-based learning. Their effectiveness challenges the assumption that differentiability is always paramount for complex control learning. An emerging direction is Unsupervised RL, which uses intrinsic objectives to allow robots to discover diverse locomotion behaviors without external rewards, pointing toward future generalist policies (Laskin et al., 2020; Ha and Schmidhuber, 2018; Oord et al., 2018). Unsupervised RL may hold the key to unlocking more general and adaptive motor intelligence. By shifting the focus from task-specific reward signals to intrinsic motivation and exploration, it opens the door to policies that can adapt to unforeseen challenges without explicit retraining. This direction aligns with broader AI ambitions toward autonomy and open-ended learning, though it remains largely experimental for locomotion tasks.

### 3.2 Other AI methods

Although non-reinforcement learning AI algorithms have not been explored as widely as reinforcement learning, there has still been notable research in this area. For instance, MPC-based neural networks (Carius et al., 2020) and other model-based networks (Viereck and Righetti, 2021) have been applied to quadruped robot control and planning.

**Figure 6:**
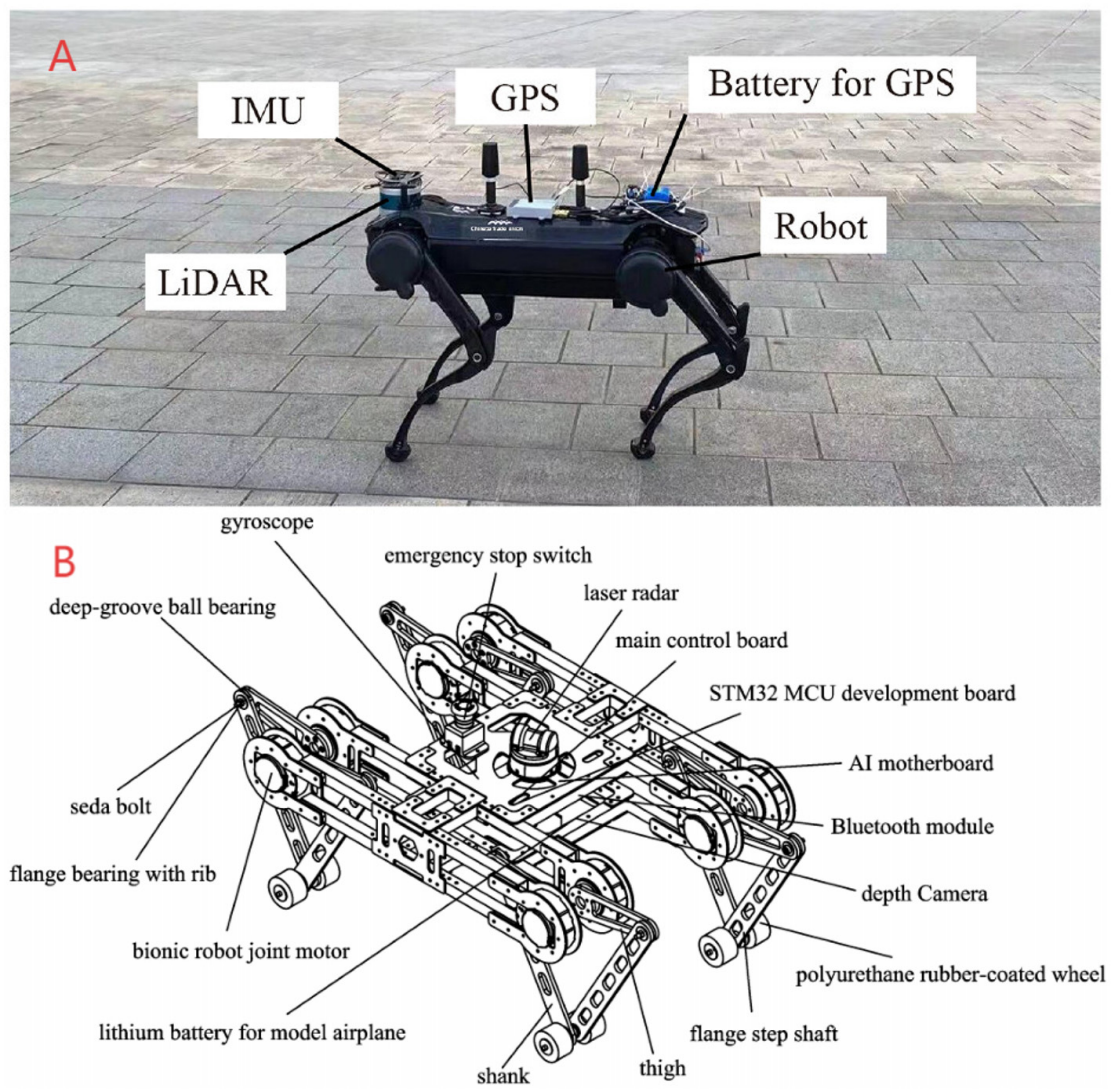
**(A)** A quadruped robot equipped with various sensors, including IMU, GPS, LiDAR, and a battery for GPS, enabling efficient control and planning. **(B)** A detailed diagram of the robot's physical structure, highlighting key components such as the bionic robot joint motor, main control board, which contribute to the robot's adaptability and performance in dynamic environments [**(A, B)** are from Zhou et al., 2024; Liang et al., 2024 and are permitted under CC4].

In the context of Identifying the physical properties of the surrounding environment, Barasuol et al. (2015) proposed a reactive trotting controller for a quadruped robot that improves foot placement using multiclass logistic regression. Magaña et al. (2019) further enhanced adaptability by combining a physics decoder with a convolutional neural network (CNN) to predict terrain parameters. Stone et al. (2020) employed an online learning approach based on Gaussian Process Latent Variable Models (GP-LVM) to interpret tactile data in real time. Villarreal et al. (2020) integrated Model Predictive Control (MPC) with CNN-driven foothold adaptation. Shi et al. (2024) used a Multi-Layer Perceptron (MLP) neural network to predict 6D contact forces based on foot deformation features. Chen et al. (2024a) proposed a cross-modal self-supervised learning framework that enables legged robots to predict terrain friction and stiffness. Additional studies related to terrain perception research are available in Kalakrishnan et al. (2011); Klamt and Behnke (2019). The work on terrain perception and adaptation is arguably where some of the most immediate practical gains are being made. While high-level DRL policies handle gait generation, these perception modules provide the essential “ground truth” awareness needed for robust traversal. The fusion of classical filtering, physical models, and neural networks here is particularly effective, demonstrating that hybrid AI architectures often outperform purely data-driven or purely model-based approaches in complex, noisy real-world settings. This perception-centered direction also connects naturally with recent language- and multimodal-AI interfaces, because a quadruped that can identify terrain, objects, or landmarks is better positioned to ground high-level instructions in the physical scene. Figure 6 shows quadruped robots equipped with various sensors for control and planning.

Another research direction is improving calibration accuracy and efficiency, Li et al. (2024c) proposed an automatic high-precision calibration method for quadruped robot legs and feet using machine vision and artificial neural networks. Although this is a relatively new direction in quadruped robotics, it offers significant potential for further research. Finally, the focus on automated system calibration addresses a critical but often overlooked bottleneck in robotics: maintenance and long-term reliability. By leveraging AI for self-diagnosis and adjustment, the field moves closer to creating robots that are not only intelligent in task execution but also in self-preservation and operational consistency, reducing the need for constant human intervention.

## 4 Advanced motion capabilities

**Figure 7:**
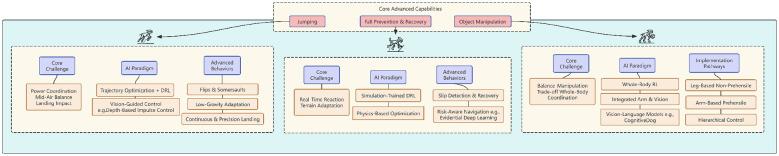
Overview of advanced motion capabilities in robotics.

Beyond straightforward walking and trotting, researchers are pushing quadrupeds to perform more dynamic and adaptive motions inspired by animal capabilities. In this section, we review three categories of advanced behaviors that have seen significant progress: jumping, falling prevention and recovery, and object manipulation. These represent milestones toward quadrupeds that can handle complex real-world tasks with autonomy and grace. Each subsection discusses the challenges of the behavior, the strategies developed, and notable experimental demonstrations from recent literature. The overview of advanced motion capabilities for quadruped robots is shown in Figure 7.

### 4.1 Jumping

**Figure 8:**
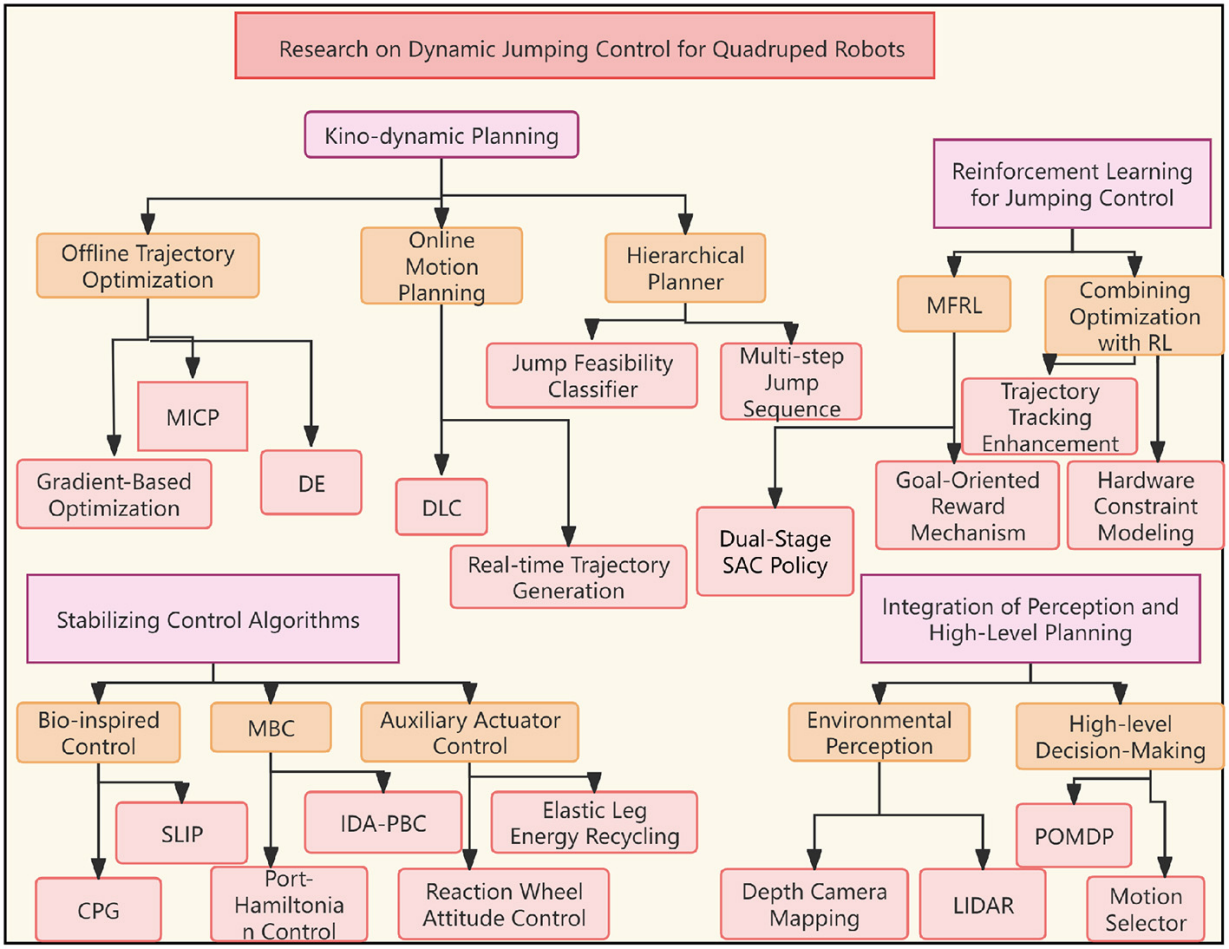
Framework of dynamic jumping control strategies for quadruped robots.

The framework of dynamic jumping control strategies for quadruped robots is shown in Figure 8.

#### 4.1.1 Trajectory optimization and motion planning

Early work on quadruped jumping applied optimal trajectory optimization methods to plan feasible jumps under dynamic constraints. Gradient-based optimizers have generated impressive leaps, such as a quadruped jumping onto a high table, but often require careful initialization and remain restricted to offline computation or planar motions. Mixed-integer convex programming has also been explored to find jump trajectories over terrain without a prior motion guess, and collocation methods have handled obstacle-clearance jumps, although these were limited to two-dimensional cases and offline planning. To overcome the limitations of slow offline solvers, researchers introduced meta-heuristic approaches. Song et al. (2022) propose an offline differential evolution optimization that finds energy-optimal jumping trajectories in full 3D without needing predefined contact schedules or reference motions. Their framework uses a hierarchical fitness function named prioritizing ground reaction force constraints to improve convergence, and it stores solutions in a motion library for reuse. Building on this, Yue et al. (2023) develop a “DLC” online planning framework combining Differential Evolution, Latin hypercube sampling, and Configuration space pruning, achieving near-real-time jump trajectory generation. By constraining the search space and using better initial samples, they report over 200 × speed improvements that enable on-board re-planning of various jumps in seconds. This evolutionary approach was validated in hardware with complex maneuvers like flips and spinning jumps executed on a Mini Cheetah robot in real time. The shift from offline to online trajectory planning marks a crucial step toward real-world applicability. The ability to re-plan jumps dynamically not only enhances adaptability but also mirrors how living organisms adjust their movements in real time.

Another direction is to embed jumping capabilities into a larger multi-step planning paradigm. Chignoli et al. (2022) present a hierarchical planner that integrates jumping into long-range locomotion plans. They formulate a fast trajectory optimizer for omnidirectional jumps that runs onboard in real time. The resulting feasible jump motions are then distilled into a low-dimensional jump feasibility classifier through learning. At runtime, a high-level sampling-based planner uses this classifier to decide when and how to incorporate jumps into routes across rough terrain. This approach ensures only dynamically feasible jumps are chosen and even ranks candidate jumps by a robustness metric to account for execution uncertainties like control error. Using onboard perception and this planner, their Mini Cheetah Vision robot reliably performed goal-directed sequences of jumps onto obstacles as tall as its hip height, vastly expanding mobility beyond planners that restrict jumps to planar motions. Such hierarchical planning showcases how trajectory optimization, combined with learned models, allows fast and reliable incorporation of jumps into field navigation. Hierarchical planning represents a sophisticated fusion of model-based optimization and data-driven learning. It embodies the trend toward more cognitive robotics, where high-level decision-making is informed by both physical constraints and learned experience.

#### 4.1.2 Control algorithms and stabilization strategies

Designing effective controllers for jump execution is crucial due to the highly dynamic and underactuated nature of jumping. Bio-inspired control has proven useful for generating robust, rhythmic jump motions. Zhang et al. (2021) develop a Central Pattern Generator (CPG)-based controller for quadruped jumping. By observing the jump sequence of a bobcat, they decompose the jump into phases and tune a network of coupled Hopf oscillators to produce the corresponding joint trajectories. The CPG network enables smooth transitions between static gaits and dynamic jumping, providing inherent stability and adaptability. In simulation, this CPG controller achieved stable periodic jumping, laying a foundation for seamlessly switching a robot between running gait and jump maneuvers. Similarly, Pi and Zhang (2024) combine multiple bio-inspired models in a unified control framework to realize continuous running jumps. They use a CPG-driven finite state machine to toggle the robot's gait between bounding and jumping modes. During bound gait, a simplified Spring-Loaded Inverted Pendulum (SLIP) model modulates leg compliance for energy-efficient running. When a jump is triggered, an offline-optimized foot-ground force profile is applied to launch the robot, and a flight-phase controller adjusts leg posture for soft landing. This fusion of CPG rhythm generation with SLIP-based thrust control allowed a simulated quadruped to execute repeated jumps at 1.75 m/s running speed, reaching a center-of-mass height of 1.22 m, and then continue running steadily upon landing. The results demonstrate that state-machine coordination of bio-inspired models can yield agile and repeatable jumping during high-speed locomotion. Bio-inspired controllers highlight an elegant synergy between biological principles and engineering design. They often provide robustness and natural motion profiles that are difficult to achieve with purely analytical controllers.

Advanced model-based control techniques have also been applied to improve jump stability. Zhang et al. (2024b) introduce a rigorous energy-based modeling and control scheme using the Port-Hamiltonian framework. They derive a full-body dynamic model of a frog-inspired jumping robot with torso torsional springs in port-Hamiltonian form, which naturally incorporates energy storage and dissipation. On this foundation, they design an Interconnection and Damping Assignment Passivity-Based Controller (IDA-PBC) that regulates the robot's joint trajectories through take-off and landing. The controller essentially shapes the system's energy landscape to ensure stable limit-cycle behavior during jumps. Simulation tests show the port-Hamiltonian/IDA-PBC approach achieves smooth and robust tracking of a desired jump motion, outperforming conventional state-feedback or sliding mode controllers in maintaining stability throughout the jump phases. Another technique for stabilization during jumping is the use of auxiliary actuators for mid-air posture control. For example, Kolvenbach et al. (2019) equip their SpaceBok robot with a reaction wheel to control pitch orientation during lunar-gravity jumps. Their jump controller actively spins the reaction wheel in flight to correct orientation errors of up to 15° and ensure upright landing. This, combined with elastic leg actuators to recycle energy, enabled repetitive 0.9-1.3 m high jumps in low gravity with improved landing accuracy and energy efficiency. Overall, these works highlight that robust quadruped jumping requires a combination of precise trajectory tracking and clever use of dynamics, such as exploiting natural compliance or added actuators, to maintain balance and minimize impact upon landing. The integration of auxiliary actuators like reaction wheels is a creative engineering solution that extends the robot's inherent capabilities. It underscores the importance of thinking beyond leg-centric control in highly dynamic scenarios.

#### 4.1.3 Reinforcement learning for jumping locomotion

Learning-based approaches have recently shown great promise in handling the complexity of quadruped jumping control, especially when modeling and manual tuning become intractable. Zhang et al. (2024a) propose a model-free DRL framework that teaches a quadruped to perform targeted forward jumps. Their architecture formulates jumping as a two-phase process including take-off and flight/landing and trains two Soft Actor-Critic (SAC) policies, one per phase, in simulation. A key innovation is the inclusion of a “target-guided” reward mechanism: the robot is given a desired jump distance, and the reward encourages moving closer to that target each attempt. This guides exploration more efficiently than unguided trial-and-error. They also integrate an intrinsic curiosity module to further improve exploration in the high-dimensional action space. The learned controller is end-to-end, mapping state estimates to joint torques without relying on any prior model of the robot. Notably, the training yielded a policy that achieves the specified horizontal and vertical jump distances with a stable landing posture, purely through learned behavior. The success of model-free RL in jumping tasks is particularly encouraging. It suggests that with sufficient exploration and well-designed rewards, robots can discover effective strategies that might not be obvious from first principles.

Beyond single jump skills, researchers have also used RL to enhance robustness and versatility across many jumping scenarios. Bellegarda et al. (2024) combine trajectory optimization with deep RL to achieve robust quadruped jumping under real-world uncertainties. They start with a physics-based jump planner and then use RL to learn a feedback policy that tracks and adapts these trajectories in the presence of disturbances or modeling errors. Instead of training from scratch, their policy augments a nominal optimal trajectory, for example, correcting body pitch if a foot launches from uneven ground or adjusting mid-flight posture if take-off was imperfect. Crucially, they incorporate hardware constraints into the simulation and reward design so that the learned policy respects the robot's actuation capabilities. This allowed them to deploy the learned controller on a real Unitree A1 robot zero-shot. The policy reliably produced jumps on hardware that were twice the robot's body length in distance, even on rough terrain, and tolerated footstep height disturbances up to 6 cm without failing. Compared to pure optimization-based control, the RL policy showed superior robustness to unknown terrain and maintained performance even when the robot's dynamics deviated from the ideal model. The hybrid approach of combining optimization with RL seems to offer the best of both worlds: the performance and safety of model-based planning, and the adaptability and robustness of learning. This is likely a key direction for future robust locomotion systems.

#### 4.1.4 Perception and high-level planning integration

To effectively deploy jumping in the real world, quadrupedal systems must perceive their environment and make deliberate decisions about when and how to jump. Modern approaches therefore tightly integrate perception modules and high-level planning with the low-level jump capabilities. For instance, the hierarchical framework by Chignoli et al. (2022) uses an onboard depth camera to build a local elevation map, which the planner uses to identify upcoming obstacles and decide if a jump is needed. Their high-level planner treats jumping as another mode of locomotion and will select a jump action if it's the only feasible way to traverse a large terrain gap or reach a higher surface. Similarly, MIT researchers (Pi and Zhang, 2024) demonstrated vision-guided autonomous obstacle jumping on the Cheetah 2 robot by using a LIDAR sensor to detect hurdles and compute the optimal take-off point before executing a jump. These systems emphasize foresight: the robot must look ahead, identify where a jump is required, and trigger the jump maneuver with proper timing and trajectory to safely clear the obstacle. The integration of perception transforms jumping from a pre-programmed trick into a contextual skill. This shift is fundamental for autonomous operation in complex environments, moving robots closer to animal-like situational awareness.

In addition to obstacle avoidance, perception can inform which jump trajectory to use from a set of capabilities. In the meta-heuristic planning work of Song et al. (2022), the authors included a motion selector that chooses an appropriate pre-optimized jump from a library based on the current scenario. The selector takes high-level inputs, from the user or perception, such as the obstacle height and distance to cross, and picks a jumping motion that matches those requirements. This speeds up decision-making during deployment and ensures the chosen jump is dynamically feasible for the given environment. Another aspect of perception is handling partial or uncertain observations. Zhang et al. (2024a) formulate their RL jumping controller as a partially observable MDP, allowing the policy to rely on proprioceptive sensors and limited exteroceptive cues to handle real-world conditions like sensory noise or terrain uncertainty. By accounting for partial observability during training, the learned policy became more robust when transferring to physical hardware or more complex environments. Formulating control under partial observability is a pragmatic acknowledgment of real-world limitations. It moves learning frameworks away from idealized simulations and toward deployment-ready robustness.

Table 5 summarizes the reported jumping performance of representative quadruped robots in the literature.

**Table 5:** Reported jumping performance in selected quadruped-robot papers.

Paper	Platform/ setting	Reported vertical metric (m)	Reported horizontal metric (m)	Other quantitative notes
An Optimal Motion Planning Framework for Quadruped Jumping	Mini-Cheetah (hardware)	Obstacle height: 0.30 (window obstacle), 0.27 (rectangle obstacle); obstacle set up to 0.35	–	Pre-motion library lookup ≈0.26 ms; includes multiple aerial skills (e.g., yaw-spin, flips).
Rapid and Reliable Quadruped Motion Planning with Omnidirectional Jumping	Mini Cheetah Vision (hardware)	Step/surface height: 0.20 (lateral), 0.22 (rotational)	–	Required aerial rotation about 60°–100° in one constrained case; planner ≈0.55 s; per-jump optimization ≈1.91 s.
Evolutionary-Based Online Motion Planning Framework for Quadruped Robot Jumping	Mini-Cheetah (hardware)	Max four-leg vertical jump height: 0.70	Desired lateral/back jump distance: 0.30	Solve times reported for multiple optimizers: 0.11 s, 0.31 s, 0.48 s, 3.49 s, 4.18 s.
CPG-Based Gait Control Method for Quadruped Robot Jumping Movement	Simulation	Max jump height ≈0.23	–	Forward speed after take-off ≈6 m/s.
Jumping Locomotion of Quadruped Robot During Running Based on Multiple Model Fusion	Simulation	COM height reaches 1.22	–	Bound gait running speed 1.75 m/s.
Robust Quadruped Jumping via Deep Reinforcement Learning	Unitree A1 (hardware + simulation)	Example goal height 0.20; robust to foot-height disturbance up to 0.06	Example goal distance 0.60; up to 2 × body length reported	Focuses on robustness under terrain variation and actuator limits.
Toward Jumping Skill Learning by Target-guided Policy Optimization for Quadruped Robots	Simulation (Aliengo)	Max vertical displacement ≈0.56 for target 0.60	Max COM displacement ≈0.47 for target 0.60	Reports target-conditioned jumping results from 0.20 to 0.60 m.
Toward Jumping Locomotion for Quadruped Robots on the Moon	SpaceBok on lunar-gravity testbed	Repetitive vertical jumps >0.90; single leap up to 1.30	–	Reaction-wheel-assisted attitude control; elasticity reduces energy consumption.
Port-Hamiltonian Modeling and Jumping Trajectory Tracking Control for a Bio-inspired Quadruped Robot	Simulation / modeling	Vertical displacement: 0.788	Horizontal displacement: 1.035	Jump duration ≈0.41 s; desired horizontal speed 2.50 m/s.

### 4.2 Falling prevention and recovery

Modern approaches to handling falls in quadruped robots leverage a range of AI-driven techniques. The goal is twofold: (i) to control or reorient the robot during an unavoidable fall to minimize impact damage, and (ii) to recover to a stable standing posture afterward. Researchers have explored reinforcement learning, optimal control, supervised learning, and hybrid strategies to address these challenges.

#### 4.2.1 Fall prediction and proactive balance control

Effective fall mitigation in quadrupeds begins with early fall prediction and robust balance control to preempt falls. Sun et al. (2024) develop a capturability-based prediction algorithm that identifies when a quadruped's state will lead to an unavoidable fall. This model-based predictor can forewarn impending falls with 95% accuracy about 0.4 s in advance. Early warning is crucial, as it allows the controller to switch modes and attempt a safe landing or recovery before a collapse occurs. Beyond outright prediction, controllers can incorporate predictive modeling to maintain stability during locomotion. For instance, model-predictive control (MPC) frameworks optimize future footholds, center-of-mass (CoM) motion, and ground reaction forces to keep the robot balanced under disturbances. Corberes et al. (2021) compare such predictive controllers and introduce a robust MPC that jointly optimizes foot placements, gait timing, CoM trajectory, and contact forces. By considering future dynamics over a time horizon, MPC-based approaches can react to pushes or terrain changes, thus avoiding falls in the first place. Another strategy for proactive balance is adjusting foot placement in real time. Sun et al. (2022) propose a dynamic gait stabilization method that predicts the appropriate foot landing position from the robot's current roll/pitch and planned gait, then modulates the foot touchdown timing and location to counteract disturbances. This foot-fall adjustment technique, validated in simulation, smooths out the impact of uneven footfalls and improves stability during trotting and after lateral pushes. In summary, early prediction and predictive balance control serve as the first line of defense, enabling the robot to either avert a fall or prepare for a controlled fall in time. Notably, Wang et al. (2024b) take this one step further by learning a high-level policy to detect unstable postures and proactively trigger a fall mitigation routine. Their “Guardians as You Fall” framework employs a supervised-learned planner that monitors the robot's state and switches control modes once the robot leaves its stability region. This mechanism acts as an intelligent “guard” that recognizes the onset of a fall and seamlessly transitions the robot from normal locomotion to a dedicated safe-falling controller before a catastrophe occurs. The concept of an intelligent “guard” policy is compelling. It represents a shift from reactive to proactive safety, embedding a form of situational judgment into the control hierarchy.

#### 4.2.2 Safe falling and mid-air landing control

When a fall becomes inevitable, the control focus shifts to falling safely: i.e. minimizing damage and achieving a favorable orientation for landing. A range of AI-driven techniques have been proposed to actively control a quadruped's posture and contacts during the fall. On the model-based side, Sun et al. (2024) introduce a contact-implicit trajectory optimization approach for fall control. Their method plans a full-body trajectory that guides the robot to either avoid toppling or, if falling, to land in a controlled manner. By incorporating uncertainty in the dynamics and terrain models, the trajectory optimizer produces smoother, more robust motions despite the discontinuous contact dynamics of a fall. This optimal fall controller can be engaged just 0.06 s after a severe perturbation and is shown to either prevent the fall or at least reduce impact velocity by a factor of three compared to an uncontrolled fall. Zuo et al. (2023) likewise emphasize trajectory optimization for falls. They formulate an extended whole-body optimal control problem that maps high-level fall strategies to joint trajectories, aiming to minimize impact forces when the robot drops from mid-air. In simulation, their optimized “aerial fall” trajectories allowed a humanoid to survive a 1.5 m, 45° pitched fall with excellent shock absorption, and physical tests confirmed reduced impact damage using the planned motions. Trajectory optimization for falling demonstrates that even in failure scenarios, optimal control theory can significantly improve outcomes. It turns a chaotic event into a planned maneuver.

In addition to offline optimization, online/reactive strategies have been explored for mid-air posture control. Roscia et al. (2023) propose an optimization-based reactive landing controller that continuously updates the quadruped's leg positions during free-fall. Their controller relies only on proprioceptive sensing and an estimate of the CoM horizontal velocity. Using a Variable-Height Spring-Loaded Inverted Pendulum model, the algorithm recalculates optimal foot placement in real time as the robot is falling. This ensures that by the time ground contact occurs, the feet are oriented to catch the robot in a stable stance.

An alternative approach to safe falling is to deliberately drive the robot into a controlled fall posture, inspired by how animals like cats or parkour athletes roll to dissipate impact. Wang et al. (2024b) pioneer this idea in their “Guardians as You Fall (GYF)” framework. GYF introduces a hierarchical learning-based controller that, upon detecting instability, actively tumbles the quadruped into a safer configuration before impact. Specifically, they define three “stable modes” for a quadruped: normal upright stance, a “regular” mode, and a “reversed” mode. When a large disturbance or erratic motion threatens to topple the robot, GYF's high-level policy triggers a transition controller that rapidly guides the robot from an unstable pose into one of the stable modes by rolling or twisting as needed.

Beyond leg-only strategies, some researchers leverage additional degrees of freedom to manage falls. Ma et al. (2023b) consider a legged mobile manipulator and show the arm can be used to arrest a fall and orient recovery. While adding hardware per se is outside our scope, their control approach is fully learning-based and merits mention. They train a single policy via DRL to coordinate the robot's legs and arm during a fall. This policy learns to deploy the arm to brace or push against the ground, thereby reducing impact forces, and then leverage the arm to help right the robot.

It is also worth noting bio-inspired strategies for mid-air reorientation. Charlet and Gosselin (2022) explore cat-righting reflex maneuvers for a free-falling quadruped. They develop two theoretical frameworks and demonstrate via simulation that a quadruped with only 9 actuated degrees of freedom can achieve controlled rotations in both roll and pitch while in free fall. In other words, by swinging its legs in closed-loop “zero angular momentum” trajectories, the robot can reorient its body to land feet-down or at least right-side up. This capability to adjust orientation about multiple axes is analogous to a cat twisting to land on its feet. While Charlet's work is analytic, others have achieved similar mid-air righting using learning. For instance, Qi et al. (2024) specifically tackle the challenge of a quadruped falling in the irregular, weak gravity of asteroids. They propose a model-free RL controller for mid-air reorientation and landing that is trained in simulation and successfully transferred to a real robot in a gravity-offload testbed. Domain randomization and policy transfer techniques were used to bridge the “reality gap,” yielding a controller that could reliably orient the robot for touchdown under asteroid-like conditions. Mid-air reorientation, especially under non-Earth gravity, is a striking example of how robotics research is pushing into extreme environments. The combination of biomechanics inspiration and modern learning techniques is particularly powerful here.

#### 4.2.3 Post-fall recovery and self-righting

Despite the best preventive and mid-air control measures, falls will sometimes happen. A crucial aspect of quadruped resilience is therefore the fall recovery: getting back on the feet after a crash. Traditional approaches to self-righting often relied on carefully sequenced motions or optimization with predefined contact points. However, these can be brittle and are difficult to execute on hardware in real time. Recent works instead leverage reinforcement learning to acquire robust recovery behaviors. For example, Sun et al. (2024) implement a model-free deep RL policy for fall recovery once the robot has come to rest. Rather than assuming specific limb contact points or a fixed sequence of rolls, their RL policy learns to exploit any contacts available to efficiently right the robot. The authors divide the recovery task into two phases and design a reward scheme that guides the policy through these stages. The learned controller observes the robot's orientation and joint states, and outputs target joint positions for the legs. RL-based recovery policies exemplify the advantage of flexibility over pre-scripting. They enable robots to adapt their recovery strategy to the specific context of the fall, much like an animal would.

In the case of arm-equipped quadrupeds, Ma et al. (2023b) show that recovery and fall-mitigation can be unified in one policy. As noted, their RL agent not only softens the fall but also uses the arm to push the robot back to its feet. The outcome is an almost complete recovery success rate even for challenging initial states.

Other recent work has adopted a modular learning approach to post-fall recovery. In the GYF framework (Wang et al., 2024b), after the safe-falling controller brings the robot into a protected posture, a dedicated recovery controller is triggered to execute the stand-up motion. This recovery policy is also trained with DRL and shares a similar observation/action structure with the fall controller. Essentially, once the robot has landed in the “reversed” supine mode, the recovery policy kicks in to roll the robot to its feet. By training these behaviors in simulation with extensive randomization, GYF ensures that the quadruped can not only survive the fall but also promptly resume upright locomotion. Similarly, Qi et al. (2024) validate their asteroid-landing RL controller both in simulation and on real hardware: the robot, when dropped in a low-gravity simulator, lands on its feet oriented correctly and thus essentially “recovers” immediately upon contact. This blur between safe landing and quick recovery is an ideal outcome, which means the robot avoids a sprawling crash altogether and can keep operating. The ideal of “falling into recovery” is a powerful design goal. It reframes a fall not as a failure mode, but as a transient state within a continuous operation cycle.

A deployment-oriented fall-recovery system should also be evaluated from the perspective of mechanical safety. In addition to whether the robot returns to a standing posture, recent studies have begun to report quantities related to joint loading, impact reduction, recovery time, repeated-trial success, and hardware feasibility. Table 6 summarizes representative evidence from the fall-prediction, safe-falling, self-righting, and post-fall recovery literature. The comparison shows that current work has moved beyond one-time demonstrations, but repeated-fall statistics, actuator temperature, motor-current history, and post-impact degradation are still reported less consistently than recovery success rate or recovery time.

**Table 6:** Representative quantitative evidence related to impact-force management, joint-damage prevention, and repeated-fall reliability in fall prevention and recovery studies.

Study	Platform/validation	Reported values	Main relevance
Sun et al. (2024)	Quadruped; prediction + fall control	Prediction accuracy ≈95%; warning time ≈0.4 s; controller triggered ≈0.06 s after severe perturbation; impact velocity reduced ≈3 × .	Early warning; impact reduction.
Wang et al. (2024b)	Unitree A1; simulation + hardware	Maximum base acceleration/jerk reduced by ≈20–73% relative to baseline falling or recovery motions.	Impact-load mitigation.
Roscia et al. (2023)	Unitree Go1; reactive landing	Simulated free-fall recovery with horizontal velocity up to 3 m/s; hardware recovery under horizontal and angular perturbations.	Landing robustness.
Ma et al. (2023b)	Legged mobile manipulator; hardware	98.9% recovery from simulated falling states; lower base contact impulse, peak joint internal force, and base acceleration than baselines.	Joint/contact-load reduction.
Zuo et al. (2023)	Legged robot; aerial fall + physical tests	1.5 m fall; 45° pitched initial posture; optimized motion reduces impact damage in physical tests.	High-energy impact management.
Hwangbo et al. (2019)	ANYmal; hardware self-righting	Recovery from 9 random fallen configurations; example recovery < 3 s; policies used for >3 months; torque/velocity limits: 40 Nm, 12 rad/s.	Recovery time; hardware limits.
Li et al. (2024a)	Quadruped; indoor/outdoor hardware	Fall during motion up to 3 m/s; balance regained in < 1 s after fall; validated on flat ground and grassland.	Dynamic recovery speed.
Lu et al. (2025)	Go2; 2025 hardware + simulation	FR-Net: level-10 success 69.5% on rough terrains and 64.1% on beams; +21.8%/+25.3% over Vanilla PPO; 40° stairs/slopes with lateral displacement ≤ 0.8/1.2 m; 50 Hz onboard control.	Challenging-terrain reliability.
Deng et al. (2025)	KYON + Go2-W; 2025 wheeled-legged recovery	Recovery success up to 99.1% and 97.8%; joint-torque consumption reduced by 15.8% and 26.2% through wheel–leg coordination.	Repeated recovery; torque reduction.

### 4.3 Object manipulation

#### 4.3.1 Reinforcement learning for quadruped manipulation

DRL has emerged as a powerful approach to train quadrupeds for manipulation tasks that are difficult to script with conventional controllers. Recent research has focused on training quadrupeds to use their legs as manipulators for a variety of tasks. Arm et al. (2024) introduced Pedipulate, a system that replaces a dedicated arm with a learned leg-based manipulation controller. By training a deep RL policy to accurately track a target foot position, their quadruped gains a “pedipulation” controller that leverages whole-body motions for reach and robustness. Notably, the learned controller automatically discovers behaviors like shifting the stance or using a tripod gait when the foot needs to reach far.

Another reinforcement learning example is the framework by Yao et al. (2023), which combines learning with model-based insight. They propose a disturbance predictive control scheme wherein a high-level RL agent estimates the effect of an attached manipulator on the quadruped's balance, and a low-level controller compensates for it. In essence, the RL component learns to predict destabilizing forces and adjusts the robot's posture accordingly.

#### 4.3.2 Imitation learning and demonstration

While reinforcement learning is popular, imitation learning is another promising avenue for quadruped manipulation. In practice, collecting demonstrations for a four-legged robot with manipulation tasks is challenging, but researchers have explored proxies like teleoperation and human motion mapping. For example, Xin et al. (2022) describe a teleoperation technique that maps human arm motions to a quadruped's arm, allowing an operator to guide manipulation maneuvers. This approach, essentially a form of demonstration, was successfully used to validate a quadruped manipulation task. Imitation learning, especially via teleoperation, provides a valuable bridge for bootstrapping complex behaviors. It leverages human intuition to overcome the exploration challenge in high-dimensional spaces.

#### 4.3.3 Hierarchical and multi-modal learning strategies

A key theme in quadruped manipulation is the integration of multiple behaviors or modalities, such as locomotion and manipulation, into one coherent system. Learning-based methods have addressed this via hierarchical policies and multi-modal inputs. One representative study by Cheng et al. (2023) introduced a hierarchical RL framework for pedipulation that uses separate learned components for different sub-tasks. In their approach, one neural policy was trained for foot manipulation actions and another for locomotion stabilization, and a high-level behavior arbiter coordinated these low-level policies to accomplish tasks like kicking a ball and pressing a button. Hierarchical learning architectures mirror the way complex behaviors are organized in biological systems. They allow for specialization, reuse, and coordinated control, which is essential for multi-tasking.

Beyond motion hierarchy, multi-modal learning can also refer to incorporating diverse sensor modalities into the policy. Many current learning approaches for quadruped manipulation have focused on proprioceptive inputs and known target coordinates. An emerging direction is to integrate exteroceptive sensing so that the robot can autonomously perceive objects to manipulate. For example, Li et al. (2023b) demonstrate a quadruped that uses onboard vision to guide a leg in performing wall-mounted tasks. In future learning-based systems, one can imagine multi-modal policies that fuse camera inputs with the robot's joint feedback, enabling end-to-end learned perceptual manipulation. While such vision-driven learning for quadruped manipulation has not yet been fully realized in the surveyed papers, it remains a critical frontier to achieve greater autonomy. The integration of vision is the next logical step for autonomous manipulation. It will move the field from “movement execution” to “task understanding”, closing the loop between perception and action in unstructured settings.

Quantitative reporting in quadruped manipulation is still less standardized than in locomotion because different studies evaluate leg-based pedipulation, whole-body pushing, arm-assisted grasping, object positioning, or dynamic grasping under different task definitions. Even so, reported values such as grasp success rate, one-shot success, positioning error, payload, task-completion steps, and model size provide useful evidence for judging task-level maturity. Table 7 summarizes representative quantitative results from recent quadruped-manipulation and loco-manipulation studies.

**Table 7:** Representative quantitative performance reported in quadruped-robot manipulation and loco-manipulation studies.

Study	Platform/task	Success/accuracy	Positioning/payload	Efficiency/validation
Jeon et al. (2024)	Quadruped whole-body manipulation	93.6% success; 0.03 m / 5° tolerance	19.2 kg drum; 15.3 kg box; 27 kg robot	Simulation + hardware
Wang et al. (2024a)	QuadWBG; arm-mounted camera	89% one-time grasp accuracy	Workspace: floor to above-body height	Real-world grasping
Muhtadin et al. (2025)	Lite3 + OpenManipulator-X	75% grasp success; 12 trials	Small/slippery/heavy objects harder	Navigation–detection–grasping
Liang et al. (2025b)	Unitree B1 + Z1; DQ-Bench	GSR-T: 80.8/80.8/77.9/74.3%; GSR-S: 55.8/55.6/44.8/41.0%	OSSR: 53.2/53.2/41.8/38.5%	TSC: 35.45/35.24/35.27/34.32; 5.37M params
Jiang et al. (2024)	Wheeled-quadruped manipulator; 6D EE tracking	NR task success	Position error < 5 cm; rotation error < 0.1 rad	Simulation + hardware
Arm et al. (2024)	Pedipulation with robot leg	NR success rate	Foot load >2.0 kg	Hardware demonstrations

NR indicates that the metric was not reported in the cited work.

## 5 Reliability, efficiency, and interaction

For quadruped robots to move from lab prototypes to real-world actors, it is not enough to demonstrate isolated locomotion skills. Meanwhile, the robots must also be reliable, efficient, and able to safely interact with humans. This section discusses efforts to design better human-robot interaction (HRI) modalities, to improve the reliability of quadrupeds, and to enhance energy efficiency. These aspects are crucial for deploying quadrupeds in domains like industry, exploration, or assistive robotics, where downtime or unsafe behavior is unacceptable.

### 5.1 Human-robot interaction (HRI)

**Figure 9:**
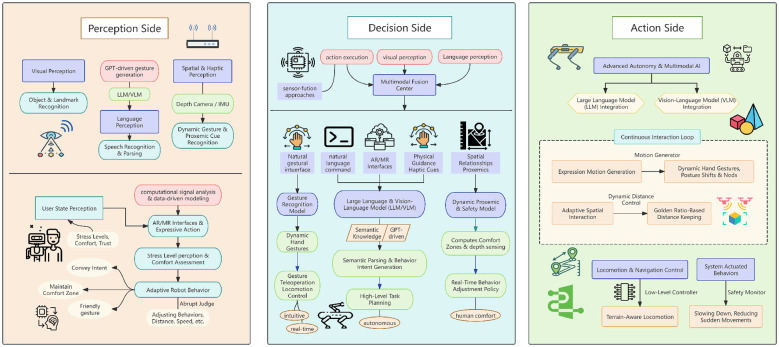
The integrated HRI framework for quadruped robots.

The evolution of quadruped robots from autonomous platforms to collaborative partners hinges on sophisticated, multi-modal interaction capabilities that are intuitive, adaptive, and socially aware. This section reviews key advancements enabling more natural and effective human-robot collaboration. The integrated HRI Framework for Quadruped Robots is shown in Figure 9.

#### 5.1.1 Dynamic gesture teleoperation

Natural gestural interfaces allow humans to command robots without physical tethers or traditional controllers. Xie et al. (2025) developed an AI-driven teleoperation system that employs deep learning-based dynamic hand gesture recognition to control a quadruped robot equipped with a robotic manipulator. In their method, a convolutional neural network (CNN) processes sensor-captured hand motion data to automatically extract spatiotemporal features and classify gestures corresponding to locomotion and manipulation commands. This AI-powered perception enables intuitive and real-time human-robot interaction (HRI), allowing operators to seamlessly control both the robot's movement and arm functions.

Complementing visual gesture recognition, haptic and physical guidance offers another intuitive interaction channel. Liu et al. (2024b) designed a human-robot physical interaction system in which a person can guide a quadruped “guide dog” robot via an inertial measurement unit based wearable or handheld device. By sensing the human's motion or force cues, the quadruped can be steered or signaled in a tactile manner, providing an intuitive way for users to interact with and control the robot through gentle physical prompts. These works collectively demonstrate a shift from explicit joystick control to implicit, natural communication, lowering the barrier for non-expert users.

#### 5.1.2 LLM-driven expressive motion generation

Large Language Models (LLMs) have recently been leveraged to enhance robot expressiveness and autonomy in interactions. Roy et al. (2025) introduced a technique to generate robot gestures and motions by prompting an LLM, effectively translating high-level textual input into corresponding expressive movements for a quadruped robot. This GPT-driven gesture generation allows the robot to convey intent or affect in response to natural language commands or dialogue. By harnessing the vast semantic knowledge of LLMs, the system can produce contextually appropriate and novel motions that were not explicitly pre-programmed. This approach has been shown to enrich HRI by making the robot's behaviors more understandable and engaging to humans. It also opens up possibilities for non-technical users to script robot behaviors using everyday language.

Building upon expressive motion generation, research is delving into finer-grained social and affective interactions. For instance, Chappuis et al. (2024) examined how a quadruped robot can perform a handshake with a human by learning personalized handshaking preferences. In their work, the robot's limb motion and force during the handshake were adjusted based on human feedback to ensure comfort and naturalness. Such research into socially intelligent behaviors, like greetings, pet-like gestures, or expressive postures, aims to make interactions with quadruped robots feel more familiar and engaging to users. The combination of LLM-driven high-level intent understanding and learned low-level social gestures paves the way for robots with nuanced social presence.

#### 5.1.3 Adaptive proxemics and spatial interaction

Proxemic behavior in human-robot interaction increasingly depends on advanced perception and adaptive control technologies. Spurny et al. (2025) introduced a dynamic proxemic model for quadruped robots that leverages deep-learning-based human detection and tracking to continuously regulate spatial relationships with nearby people. Using a stereo vision system combined with depth estimation and object-recognition algorithms, the robot perceives human position, orientation, and movement in real time. These sensory inputs are processed on an embedded GPU platform, which computes the robot's motion responses through a golden-ratio-based comfort zone algorithm and a Gaussian velocity profile. This integration of computer vision, real-time depth sensing, and motion adaptation enables the robot to maintain smooth and socially appropriate distances, such as when walking beside a person or delivering an object.

To further enhance situational awareness and safety in human environments, robust multi-sensor perception is essential. Beyond this, robust environment perception is being enhanced by sensor-fusion approaches to aid quadruped robots in safely navigating around humans and obstacles (Chen and Hong, 2023). The fusion of LiDAR, cameras, and inertial data creates a more reliable world model. This robust perception directly supports not only safe navigation but also more complex interactive tasks, serving as the foundation for the multimodal systems discussed next.

#### 5.1.4 Human stress response and perceived safety

Understanding human psychological and physiological responses to robots is essential for designing socially acceptable human-robot interactions. Gupta et al. (2024) employed computational signal analysis and data-driven modeling to examine human stress and perceived safety during encounters with autonomous quadruped robots. Their study combined multimodal physiological sensing through electrocardiography and electrodermal activity with advanced signal processing and machine learning techniques. These included feature extraction using NeuroKit2, signal decomposition through convex optimization with the cvxEDA method, and classification based on ensemble algorithms such as AdaBoost and RusBoost trained under a leave-one-subject-out cross validation protocol. This framework enabled automatic decoding of acute stress responses and identification of motion patterns that influenced human arousal levels.

Hashimoto et al. (2024) used behavior modeling and statistical learning to explore how a quadruped robot's expressive actions affect perceived safety. They programmed dominant and submissive postures via the Boston Dynamics Spot software development kit and analyzed motion-tracking data using mixed effects modeling and analysis of variance to quantify behavioral and psychological differences. This line of research is crucial for ensuring that increasingly capable and autonomous quadrupeds are not just functionally effective but are also perceived as predictable, non-threatening partners by the humans they work alongside.

#### 5.1.5 Multimodal language–vision–action systems

A frontier in quadruped HRI is the integration of language understanding, visual perception, and action execution into a unified system. Lykov et al. (2024) developed the “CognitiveDog” platform, which employs a large-scale multimodal model to translate human language instructions and visual scene information into real-time actions by a quadruped robot. In this system, the robot's onboard vision is used to recognize objects or landmarks in the environment, while a language model parses spoken or written commands from a user; the two modalities are fused to generate appropriate navigational or manipulation actions. For example, if a user says “find the red toolbox and come back,” the CognitiveDog interprets the request, visually identifies the toolbox, and plans a path to retrieve it. Such multimodal frameworks significantly improve the intuitiveness of HRI, allowing users to interact with robots through natural language and gestures grounded in the shared environment.

Augmented Reality (AR) interfaces offer a powerful medium to visualize and command this autonomy, creating a shared cognitive space between human and robot. Ulloa et al. (2023) developed a mixed-reality system for commanding and monitoring a quadruped robot in search-and-rescue scenarios. Through an AR headset, the human operator can visualize the robot's sensor data and intended path overlaid on the real world, and can issue spatial instructions, such as placing virtual waypoints or drawing gesture cues in the AR view, that the robot then follows. This approach leverages human spatial understanding and reduces cognitive load by integrating the robot's perception and intent directly into the user's field of view, creating a highly intuitive collaborative workflow.

Shared autonomy is an important bridge between direct teleoperation and full autonomy. In shared-autonomy settings, the human provides high-level intent while the robot handles local perception, balance, obstacle avoidance, and safety constraints. This requires intent prediction, trust-aware interaction, explainable AI, and safety-aware human–robot collaboration. Intent prediction allows the robot to infer whether a person wants the robot to follow, stop, yield, approach an object, or hand over control. Trust-aware interaction requires the robot to expose its confidence, planned motion, and failure modes so that humans can calibrate reliance. Explainable AI is particularly important for learned policies because operators need understandable reasons for robot actions, especially in safety-critical settings such as search-and-rescue or assistive guidance. Safety-aware HRI should combine proxemic control, contact-force limits, emergency stop behavior, and transparent communication of robot intent.

Table 8 summarizes representative quantitative evidence for shared autonomy, trust-aware interaction, intent prediction, explainable interaction, and safety-aware collaboration. These studies use different platforms and tasks, so the values should be interpreted as reported evidence rather than normalized benchmarks.

**Table 8:** Representative quantitative evidence for shared autonomy, trust-aware interaction, intent prediction, explainability, and safety-aware HRI.

Study	Focus	Platform/setting	Reported indicators	HRI implication
Gupta et al. (2024)	Trust/perceived safety	Spot + Unitree Go1; shared human space	ECG + EDA + self-report; 2 robot types; stress: encounter > baseline; multiple robots > single; navigation > search	Trust-aware motion design
Hashimoto et al. (2024)	Social safety cues	Spot; 2 × 2 within-subjects	Dominant vs. submissive; head-on vs. crossing; submissive perceived safer	Legible behavior / trust
Ulloa et al. (2023)	MR shared autonomy	Quadruped + 6-DoF arm; SAR	MR/VR comparison; task time, training, confidence, falls/collisions; MR: higher field visibility/trust; VR: remote safety	Operator awareness
Spurny et al. (2025)	Proxemics / safe spacing	Quadruped; stereo/depth perception	Human pose + distance tracking; golden-ratio comfort zone; Gaussian velocity profile	Spatially safe navigation
Sambhus et al. (2025)	Shared autonomy + CBF safety	Unitree Go2; teleoperation	ANMPC 10 Hz; NMPC 60 Hz; WBC 500 Hz; online Boltzmann intent model; user study	Safety-aware shared control
Contreras et al. (2025)	Intent prediction	Mobile manipulation; 5 users × 5 tasks	25 trials; navigation stability 93–100%; manipulation 94–100%; intent prediction 23.6 s vs. 7.8 s baseline	Early intent inference
Liu et al. (2025)	Intent-aware adaptive HRC	Vision–language–force–state HRC	CVAE + Transformer; multimodal intent estimation; IROS 2025; quantitative values NR	Mode switching / explainability

NR indicates that the value was not reported.

### 5.2 Fault diagnosis and recovery

Legged robots are complex systems with many potential failure modes: a joint motor could fail or jam, a sensor could malfunction, or structural damage could occur. Nature offers inspiration: three-legged dogs learn to walk, and animals can compensate for injuries to some extent. Researchers aim to imbue quadrupeds with similar fault tolerance. Various approaches have been developed to enhance fault detection and recovery mechanisms.

Support Vector Machines (SVM) have been employed for trajectory optimization and gait pattern recognition, leveraging their ability to handle high-dimensional input spaces effectively (Liling et al., 2015). Expanding on this concept, Xu et al. (2016) presented a fault diagnosis framework that integrates Information Entropy (IE), Relevance Vector Machine (RVM), and a Gaussian-disturbed Cuckoo Search algorithm (GCS). These traditional machine learning methods provide a solid, interpretable baseline for fault classification, though they may struggle with the high-dimensional, temporal nature of fault progression in dynamic systems.

Beyond traditional machine learning techniques, learning-based fault diagnosis has also gained traction. Ma et al. (2023a) introduced a Noise-Excitation Generative Adversarial Network (NE-GAN) tailored for actuator fault diagnosis in multi-legged robots. Additionally, Wang et al. (2025) proposed a hybrid deep neural network architecture for fault diagnosis, combining Convolutional Neural Networks (CNN), Gated Recurrent Units (GRU), and attention mechanisms. The shift to deep learning, particularly with hybrid architectures, represents a significant advance, enabling the model to autonomously extract spatio-temporal features from raw sensor data that are indicative of incipient faults. DreamFLEX (Lee et al., 2025) is a notable example of a learning-based fault-aware locomotion controller. More related research can be seen in (Zong et al., 2024) and Chen et al. (2024b).

While current systems can handle single-point failures quite well, handling multiple simultaneous faults or more subtle degradations remains an area of active research. This is the next frontier: moving from graceful degradation under a known failure to robust survival under compound, uncertain, and partial failures, mirroring the robustness of biological systems in truly unpredictable scenarios.

### 5.3 Energy efficiency

Energy efficiency is a crucial factor for mobile robots, directly affecting operational endurance, payload capacity, thermal safety, and battery utilization. A quantitative energy discussion should distinguish several metrics: instantaneous electrical power, mechanical work, energy per traveled distance, cost of transport (CoT), battery state-of-charge change, and energy consumed per task. AI strategies help improve efficiency by incorporating one or more of these measurements into the control objective. In reinforcement learning, this commonly means adding penalties for joint torque, joint velocity, electrical power, or battery drain while maintaining constraints on tracking, speed, and stability. However, excessive energy penalties can slow the gait or reduce robustness, so energy-aware control is inherently a multi-objective trade-off.

Recent work has looked at adapting gait parameters for efficiency. Hao et al. (2024) proposed a hierarchical controller where a high-level RL policy adjusts the stance ratio in real time to optimize energy use. The Policy Search Transfer Optimization (PSTO) method (Zhu and Rosendo, 2022) combined deep RL and classical optimization to yield energy-efficient locomotion. Yan et al. (2024b) introduced a DDPG-based approach to minimize energy consumption in the Cyber Dog quadruped robot. Sulpice et al. (2025) proposed the FootStep Reward to encourage energy-efficient gait patterns, while Wei et al. (2024) developed a gait-heuristic reinforcement-learning framework for smooth and energy-aware locomotion. These studies illustrate that energy efficiency is improved not by a single algorithmic component, but by the combined design of reward terms, gait parameters, actuator usage, terrain adaptation, and stability constraints.

Reported energy results are not fully standardized across quadruped platforms, but several studies provide useful numerical evidence for comparing power consumption, CoT, battery-related performance, robustness, computation, and hardware constraints. Table 9 summarizes representative values. The table also shows why energy efficiency should be interpreted together with success rate, terrain robustness, actuator limits, battery endurance, and thermal load, rather than as an isolated reward term.

**Table 9:** Representative quantitative evidence on energy efficiency, robustness, computation, and hardware constraints in AI-driven quadruped locomotion.

Study	Energy/battery metrics	Robustness/performance metrics	Hardware or control constraints
Hwangbo et al. (2019)	Torque: 8.23 vs 11.7 Nm; power: 78.1 vs 97.3 W	Speed: 1.5 m/s; tracking error: 0.143 m/s; yaw error: 0.174 rad/s; self-righting: < 3 s	ANYmal hardware; actuator-model RL; torque and velocity constraints
Lee et al. (2020)	CoT: 0.423 / 0.692 / 1.23 on moss / mud / vegetation	Payload: 10 kg; step: 13.4/16.8 cm; DARPA test: 4 × 60 min, zero locomotion failure	ANYmal hardware; proprioception + exteroception; rough-terrain policy
Yan et al. (2024b)	Energy: 1.6236 / 1.8152 / 2.8261 kJ; saving: 12 / 11 / 9%; range: 3600–3924 m	Speeds: 1.0/1.2 / 1.4 m/s; sim + prototype validation	CyberDog; DDPG energy optimization; 32 Nm motors; 220 rpm; Jetson Xavier NX
Liang et al. (2025a)	Distance-averaged energy reward; energy weight α_*en*_ = 1.0	Speed range: 0.1–2.5 m/s; step: 20 cm; sim + Go1 hardware	Unitree Go1; single policy; adaptive gait transition; reward-weight tuning
Schperberg et al. (2025)	CoT improvement: 50.4% vs model-free RL	Slippery surface, gait transition, disturbance tests; sim + A1 hardware	Online planner; placement-set footstep rule; speed-dependent parameters
Mahankali et al. (2025)	Similar or lower energy than PPO baselines	Peak speed: 2.86 m/s sim; 2.5 m/s hardware; +0.74 m/s vs PPO; 1000 episodes / 3 seeds	Unitree Go1; EIPO; torque-speed clipping: 33 Nm; minimal reward shaping
Fadini et al. (2024)	Lower power than hand-tuned heuristics; design improvement: ≥52% per task	Bounding / backflip trajectories; real prototype replay	Co-design; actuator friction, torque, and bandwidth limits included

NR indicates that the metric was not reported in the cited work.

For cross-study comparison, future papers should report speed, terrain type, payload, battery condition, average and peak power, CoT or energy per meter, computation rate, and whether the controller respects actuator temperature and torque limits. Hardware limitations are especially important: actuator power density limits explosive motions, battery capacity constrains mission duration, and thermal effects can reduce torque availability during repeated high-load maneuvers. Energy-efficient policies must therefore be evaluated together with hardware safety and robustness, rather than only by simulated reward values.

By optimizing gait parameters, exploiting passive dynamics, and balancing multiple objectives, quadrupeds have become more energy-aware. Future work should integrate energy efficiency with task performance, ensuring that robots accomplish navigation, manipulation, and interaction goals in a power-aware and thermally safe manner.

## 6 Challenges and future directions

Despite remarkable progress in AI-driven quadruped robotics, significant challenges remain on the path to truly animal-like mobility and autonomy. In this final section, we discuss some open issues and future research directions that have emerged from the literature review and from the current limitations of state-of-the-art systems.

### 6.1 Further closing the sim-to-real gap

While domain randomization and related techniques have enabled zero-shot sim-to-real transfers for basic locomotion, the gap widens when it comes to more complex behaviors and changing environments. Future work will likely involve online adaptation where the robot continuously updates its model of the world and simulator improvements. Specifically, advanced RL and DRL research could focus on developing dynamic domain adaptation algorithms that utilize real-world streaming data to iteratively refine the simulation parameters or the policy itself. Meta-reinforcement learning frameworks that learn a prior over simulation dynamics or a set of adaptive policies for different environmental conditions are promising directions.

### 6.2 Integration of perception and locomotion

So far, a lot of the learned locomotion assumes either a structured environment or uses proprioceptive feedback only. Real animals rely heavily on vision to plan footholds and routes. Quadrupeds are just beginning to integrate vision and other exteroceptive sensors deeply into their locomotion controllers. We foresee future research on multi-modal sensor fusion so the robot has a rich understanding of its surroundings and can plan complex maneuvers. Ensuring that learned locomotion policies remain stable under real sensor delays and noise is a challenge; work on more advanced sim-to-real includes modeling sensor noise and using techniques like privileged training to guide policies that ultimately use noisy infomation.

### 6.3 Generalization and multi-task learning

Many of today's controllers are specialized, one for walking, one for recovery, one for manipulation, etc. Ideally, a single quadruped should seamlessly handle multiple behaviors or tasks, switching as needed. An exciting future direction is multi-skill integration. Future quadrupeds might have a skill library that they can draw from on demand. This requires advances in high-level decision making. Meta-learning might allow the robot to learn new tasks quickly by leveraging skills it already has, leading to a form of lifelong learning on the robot. Hierarchical reinforcement learning (HRL) is a key framework for this challenge. Future work includes developing more efficient and stable HRL algorithms for continuous control, skill discovery methods that autonomously build a reusable skill library, and goal-conditioned policies that provide a unified interface for diverse tasks. Furthermore, investigating large-scale multi-task and foundation model training in simulation to produce versatile, pre-trained locomotion “brains” is a promising frontier.

### 6.4 Sample efficiency and real-world training

Despite improvements, training sophisticated behaviors in simulation can still take billions of steps, and fine-tuning on real hardware is extremely limited by time and wear. Algorithms that are more sample-efficient will be important to learn complex behaviors within reasonable time. Furthermore, as real-world deployment increases, we expect more on-robot learning: e.g., a robot that improves its gait over months of operation through occasional self-calibration runs. Ensuring safety during such real-world learning is a challenge, so techniques like constrained RL and safe exploration will be crucial. The future might involve cloud-based training where many simulated instances of a robot learn in parallel, and the best policies are periodically tested and improved on actual robots in a feedback loop. Research directions include advancing hybrid methods that combine deep learning with classical control for better initialization, developing more effective exploration strategies for sparse-reward locomotion tasks, and creating frameworks for simulation-informed Bayesian optimization for safe, data-efficient real-world policy refinement.

### 6.5 Scaling to extreme environments

One frontier is deploying quadrupeds in environments too dangerous or remote for humans or other planets. Each environment adds challenges: extreme temperatures, radiation, low gravity, etc. For planetary exploration, energy and communication constraints are huge, and the robot might need to make decisions largely on its own and cannot afford wasted energy. This pushes the need for ultra-reliable autonomy and very efficient locomotion. This presents unique challenges, motivating research into energy-optimized locomotion policies trained with cost functions that explicitly minimize energy consumption or maximize travel distance. It also necessitates the development of robust RL algorithms capable of generalizing to unseen physical dynamics and capable of long-horizon, risk-aware planning under severe uncertainty with minimal human intervention.

In summary, the future of AI-driven quadruped robots is bright and multi-faceted. On the one hand, continued algorithmic innovations will make these robots more agile, versatile, and independent. However, integration with human factors and safety will make them more practical and accepted in everyday scenarios. The ultimate vision is a quadruped robot that can go anywhere a four-legged animal can, perform useful work or accompany humans, and do so autonomously with minimal oversight. Given the rapid progress of the last decade, AI-driven technologies bring us steps closer to legged robots becoming an ubiquitous extension of human capability in the real world.
